# Challenges of MS‐based small extracellular vesicles proteomics

**DOI:** 10.1002/jev2.70020

**Published:** 2024-12-18

**Authors:** Daniel Fochtman, Lukasz Marczak, Monika Pietrowska, Anna Wojakowska

**Affiliations:** ^1^ Institute of Bioorganic Chemistry Polish Academy of Sciences Poznan Poland; ^2^ Maria Sklodowska‐Curie National Research Institute of Oncology Gliwice Poland

**Keywords:** extracellular vesicles, mass spectrometry, proteomics, reliability, sEV isolation

## Abstract

Proteomic profiling of small extracellular vesicles (sEV) is a powerful tool for discovering biomarkers of various diseases. This process most often assisted by mass spectrometry (MS) usually lacks standardization and recognition of challenges which may lead to unreliable results. General recommendations for sEV MS analyses have been briefly given in the MISEV2023 guidelines. The present work goes into detail for every step of sEV protein profiling with an overview of factors influencing such analyses. This includes reporting and defining the sEV source and vesicle isolation, protein solubilization and digestion, ‘offline’ and ‘online’ sample complexity reduction, the analysis type itself, and subsequent data analysis. Every stage in this process affects the others, which could result in different outcomes. Although characterization and comparisons of different sEV isolation methods are known and accessible and MS‐based profiling details are provided for cell or tissue samples, no consensus work has been ever published to describe the whole process of sEV proteomic analysis. Reliable results can be obtained from sEV profiling provided that the analysis is well planned, prepared for, and backed by pilot studies or appropriate research.

## 1 INTRODUCTION

**FIGURE 1:**
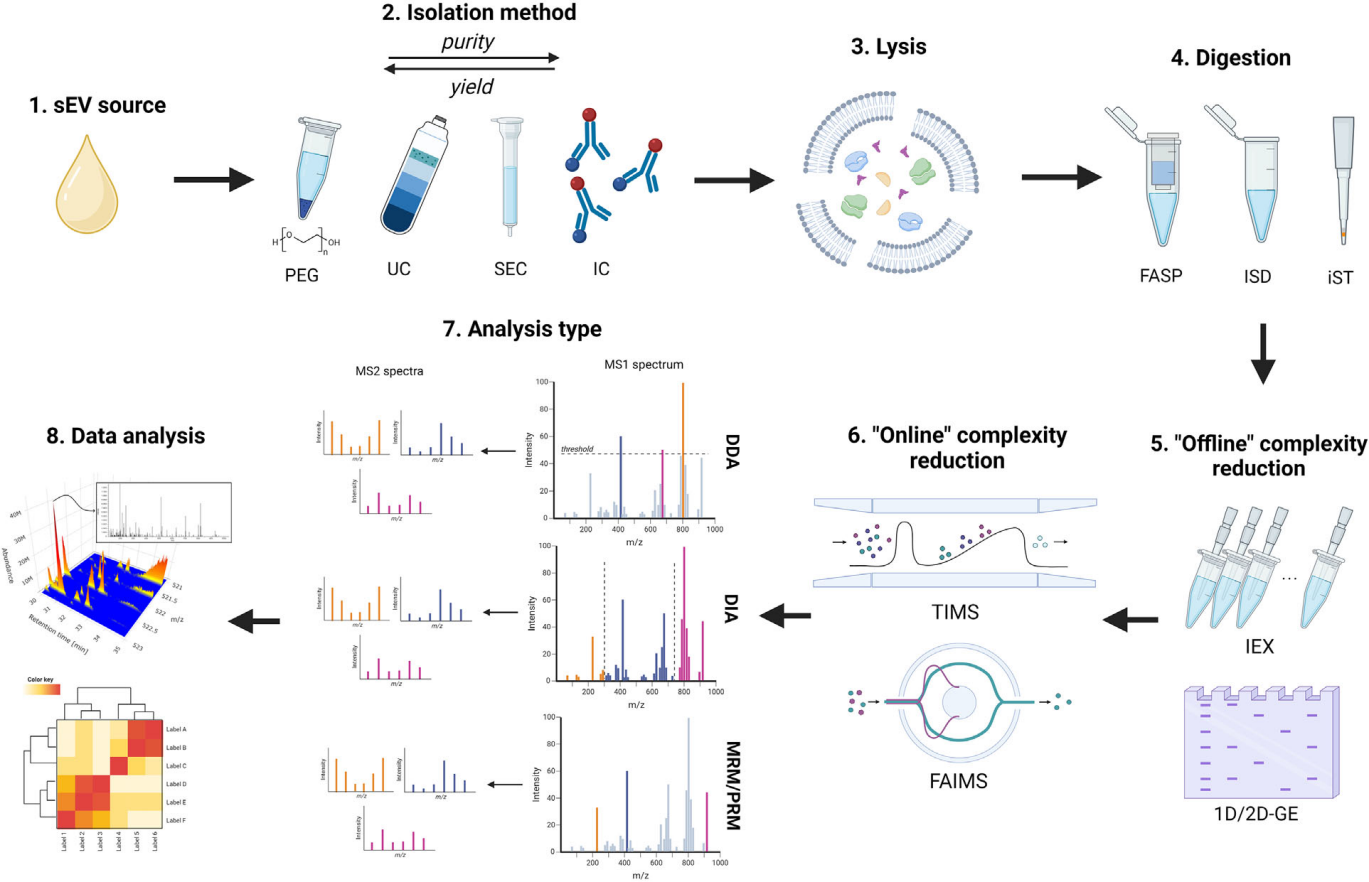
Selected factors influencing the outcome of MS‐based proteomics analysis of small extracellular vesicles. The abbreviations are polyethylene glycol‐based isolation (PEG), ultracentrifugation (UC), size exclusion chromatography (SEC), immunocapture (IC), filter‐aided sample preparation (FASP), in‐solution digestion (ISD), in‐StageTip digestion (iST), ion exchange chromatography (IEX), 1D/2D electrophoresis (1D/2D‐GE), trapped ion mobility spectrometry (TIMS), field asymmetric‐waveform ion mobility spectrometry (FAIMS), data‐dependent acquisition (DDA), data‐independent acquisition (DIA), multiple/parallel reaction monitoring (MRM/PRM).

Small extracellular vesicles (sEV), which include endosome‐derived exosomes (Welsh et al., 2024), are membrane‐bound particles lower than 200 nm in size, enriched in proteins, RNA, and other bioactive molecules. Their main function is to mediate cellular communication, as when released they transport their cargo to other distant cells (Kalluri & LeBleu, 2020; Welsh et al., 2024). Proteomic analyses of sEV have become increasingly widespread as a tool for searching for new molecular biomarkers of disease, especially in cancer, cardiovascular, and neurodegenerative diseases or transplant rejection (Alvarez et al., 2013; Giri et al., 2010; Gołębiewska et al., 2021; Makler & Asghar, 2020; Mathew et al., 2021; Moreira‐Costa et al., 2021; Saravanan et al., 2023; Vallejos et al., 2023; Wong & Chen, 2019). Due to the number of published studies analysing vesicles, a need to standardize measurement methods becomes more pressing. Moreover, it should be recognized that mass spectrometry (MS) based proteomic profiling of sEV often proves difficult, since a limited amount of material is available compared to whole cells or tissues. Although multiple studies detailing isolation and sample preparation methods have been published, certain aspects of MS‐based sEV analyses, such as protein solubilization or MS data analysis, remain poorly described. Thus, this work aims to provide a comprehensive overview of factors influencing the outcome at every stage of such analyses (Figure 1) including sEV isolation, sample preparation, MS profiling, and subsequent data analysis.

## 2 PROTEOMIC COMPOSITION OF SEV

**FIGURE 2:**
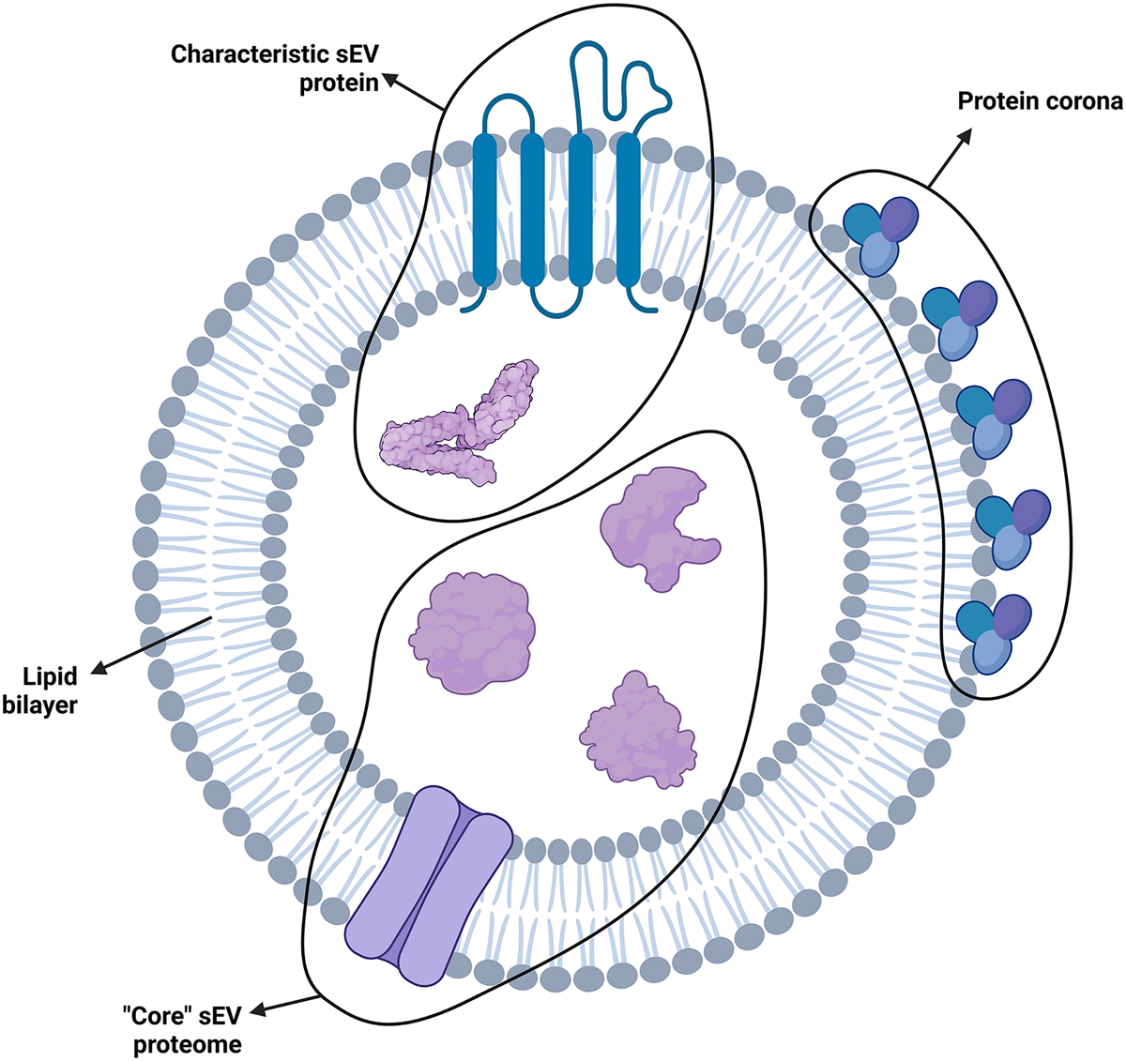
A schematic representation of the sEV proteome. It broadly consists of characteristic sEV protein (e.g., lumen protein—ALIX or transmembrane protein—CD63), protein corona (e.g., serum albumin) and the ‘core’ sEV proteome which can reflect the status of the cell of origin.

Before any protein profiling is performed one should be aware that sEV proteome consists of ‘core’ protein reflecting for the cell of origin, but also characteristic sEV protein and its corona (Figure 2). The carryover of the cell‐of‐origin proteome in the ‘core’ sEV proteome has been directly shown in vitro and in vivo models before (Frankenfield et al., 2022). Here, the most promising candidates for disease biomarkers can be identified. On the other hand, sEV also contain their own specific markers such as CD63, CD81, CD9, TSG101 which are upregulated in vesicles as compared to cells or tissues due to their biogenesis. Moreover, sEV which circulate in body fluids also carry on their surface additional proteins which make up vesicles’ corona, for example, serum proteins, which are becoming a novel aspect in sEV research (Heidarzadeh et al., 2023; Wolf et al., 2022). Due to the protein‐rich environment in which sEV reside, for example, blood serum with its albumin, vesicles can become coated with such protein. Recent study reported that 87% of corona protein abundance was comprised of anti‐thrombin III, complement C3, factor V, fibronectin, IgG, and complement factor H (Heidarzadeh et al., 2023; Zhang et al., 2020). This corona does not constitute the core proteome of the sEV, which is comprised of proteins found in the lumen of the vesicle as well as membrane‐bound proteins such as tetraspanins. Nonetheless, the protein corona may modulate the function of the vesicles, for example, their angiogenic and immunomodulatory characteristics (Wolf et al., 2022). Importantly for this overview, certain isolation methods may presumably yield vesicles with different corona makeup or abundance when compared to other techniques. Research on the performance of isolation methods with regards to the protein corona is yet to be published. With limited information, one should be aware that during MS proteomics analysis this protein may be detected and that could impact the outcome of the analysis. Moreover, due to the presence of highly abundant corona proteins, MS analyses of sEV in data‐dependent acquisition (DDA) mode may be highly impacted. This is because of the problem with the measurement of redundant MS^2^ spectra, which is described in detail further. In the end, reliable detection and quantification of low abundant, surface and cargo proteins become difficult.

One has to decide if such corona should be considered part of the vesicle as a whole or if it should be regarded as a contaminant. Depending on the approach, this part of the sEV proteome may or may not provide additional information to the presented problem. It should be noted that, their presence may be highly dependent on the vesicles’ environment and the process of protein ‘sticking’ to sEV may be at least partially random. Although some information can be gleaned from this part of the proteome (e.g., in sEV and their immunology) it is inadvisable to use such protein as a standard prognostic factor. In such case the heterogeneity of body fluids’ composition would limit the reliability of the whole diagnostic process. If the ‘core’ proteome is to be investigated and used as the source of biomarker proteins, presence of the other part of the sEV proteome imposes a requirement of high dynamic range to obtain sufficient profiling depth. Optimization of the workflow again proves to be important, since techniques such as complexity reduction or IMS can alleviate this problem.

Even with the best practices in proteomics research, sEV stand as a challenging material to analyze due to their complex biogenesis. The novel aspect of sEV protein corona highlights the characteristics of highly sensitive MS analyses—all the protein, either the core protein of the vesicle or its corona, will most likely be detected. It is not yet clear whether this corona plays a crucial functional role or if it may be seen as contamination. Regardless, sEV contamination with, for example, highly abundant serum proteins brings another technical challenge to their analysis. New methods of complexity reduction, such as ion mobility separation, as well as already well‐established methods (ion exchange chromatography, etc.) provide possible ways to deal with this problem. Yet, the impact of such methods specifically for sEV is not known.

Straightforward analysis of sEV, although challenging, should not be performed without the understanding of the biological context of the analyzed material. MS‐based proteomics should consider the biogenesis, function and makeup of the vesicles. Translation of techniques used for cells or tissues is usually performed, but may lead to suboptimal and undesirable results. Even different sEV isolation methods have been shown to yield completely incomparable populations of vesicles. Thus, only if all the above would be standardized and optimized, sEV MS‐based proteomics may be successfully used in clinical and diagnostic settings in the future.

## 3 SEV ISOLATION

Even before the sEV may be isolated, one should recognize that sample collection methodology has a resounding impact on the result of the MS‐based protein profiling. In case of clinical samples such as urine or serum, patients’ water uptake should be controlled and monitored. The importance of this has been shown for urinary exosomes, where patient water intake was tightly correlated with the number of sEV isolated from the same volume of a sample (Blijdorp et al., 2021). Moreover, after water loading, the amount of highly abundant uromodulin was increased and the characteristic sEV protein (CD63, TSG101, CD9, ALIX, CD81) levels were lower. This could be presumably explained by sample dilution, which when not accounted for, will introduce another variable into the experiment. Since high heterogeneity of ex vivo samples are expected, sEV dilution should be controlled and normalization approaches (e.g., creatinine levels in urine) may need to be used even at this initial stage. For sEV isolated from cell culture media, when an equal amount of starting material is used for all conditions, this may usually suffice as a normalization measure. Regardless of the source of sEV, contamination of the starting material should be avoided. In the case of sEV isolated from cell culture media, introduction of exogenous sEV from fetal bovine serum (FBS) may yield erroneous results (Lehrich et al., 2021; Urzì et al., 2022). In the case of blood serum, limiting and monitoring the amount of albumin may be necessary. The same can be stated about uromodulin in urine, which was mentioned above. Generally, regardless of the isolation method, the material used to obtain sEV should be well characterized beforehand and if possible—standardized to avoid contamination and initial disproportion in the number of sEV at this stage.

Vesicles that meet the criteria of *Minimal Information for Studies of Extracellular Vesicles*, that is, MISEV guidelines can be isolated using various techniques (Welsh et al., 2024) characterized by different efficiency and final sample purity (Brennan et al., 2020; Cho et al., 2020). In case of MS analyses the number of co‐isolated contaminants should be especially noted. One of the described methods uses polyethylene glycol (PEG) mediated sEV precipitation. Here PEG is used to internalize the intended particles, but on the other hand, is considered as a known MS contaminant. It produces obscuring MS^1^ spectra with peaks separated by 44 Da (Ahmadi & Winter, 2018; Rardin, 2018) making this method unsuitable for sEV MS‐based proteome profiling. Another technique, ultracentrifugation (UC), uses large forces to separate particles based on their density. This approach can be characterized by contaminating protein co‐isolation since protein aggregates, for example, ribonucleoproteins have a similar density as sEV (Yang et al., 2020; Zarovni et al., 2015). Density gradient ultracentrifugation (dUC) can address the shortcomings of regular UC. Here a solution of a specific density is used to separate vesicles based on their size. At last, even methods based solely on the size are not perfect and a certain amount of contamination should be expected. In this case, particles of the same size as sEV, for example, lipoproteins, may be co‐isolated (Sódar et al., 2016; Théry et al., 2006). Similarly, another technique based on this separation parameter is size exclusion chromatography (SEC), which suffers from the same problems as dUC, that is, lipoprotein co‐isolation. Avoiding PEG, protein aggregate, and lipoprotein contamination is possible when immunoaffinity methods are used to separate vesicles (Skoczylas et al., 2024). On the other hand, achieving an acceptable yield of immunocaptured sEV may be difficult, especially when dealing with clinical samples/material. Moreover, an appropriate antigen must exist on the surface of the vesicle for the immunocapture approach to be effective. This antigen must be specific to sEV (preferably a membrane protein) and should not be present as a soluble component of the raw sample, for example, whole urine or serum. Since sEV are defined by size, this approach alone may be insufficient and would have to be preceded by another method of size‐based isolation like dUC or SEC, which would additionally limit unspecific binding of antibodies to other antigens present in the sample. Therefore, to achieve a non‐contaminated sEV population one may employ a combination of techniques beyond SEC and immunocaputre, albeit with the possibility of a limited yield. To summarize, isolation methods usually balance the amount of vesicles/protein that can be isolated, with the final sample purity and/or time required for isolation. It means that a method which results in a high number of sEV, may not isolate them with acceptable purity, which is the case for example with PEG‐mediated methods. On the other hand, techniques such as immunocapture which allow the isolation of strictly defined particles, may yield only a very limited amount of material for the subsequent analysis. Thus, methods which keep a good balance of yield and purity, such as dUC and SEC, are most often used in practice. Performing an optimization study before the actual experiment may be recommended when possible.

## 4 SEV SAMPLE PREPARATION

### 4.1 Solubilization of sEV proteins

Before profiling with mass spectrometry in a bottom‐up approach, proteins in each sample must be enzymatically digested into peptides, which make‐up such proteins. The proteome of sEV can be analyzed only when their cargo proteins have been released from the vesicle and solubilized, thus allowing their digestion. This process is carried out using solution or buffers, which contain ingredients such as detergents, salts, chaotropic agents, reducing agents, protease and/or phosphatase inhibitors, and pH‐stabilizing agents. The role of each component should be recognized when choosing a lysis buffer, especially since it may impact the subsequent analysis due to poor protein digestion, MS compatibility, or ion suppression effects.

Detergents such as sodium dodecyl sulfate (SDS), sodium deoxycholate (SDC), or Nonidet P‐40 (NP‐40) allow the breakup of the vesicle membrane and solubilization of released proteins with the help of added inorganic salts—most commonly sodium or potassium chloride. The choice of detergents is especially important since their presence in a sample suppresses electrospray ionization (ESI) used in mass spectrometry analyses (Ilavenil et al., 2016; Quirino, 2018). Certain preparation techniques, for examle, filter‐aided sample preparation (FASP) (Wiśniewski, 2018) or single‐pot solid‐phase‐enhanced sample preparation (SP3) (Hughes et al., 2019) have been developed to remove detergents before MS analysis. It is also possible to use detergents compatible with ESI like RapiGest (Pop et al., 2014), SDC (Scheerlinck et al., 2015), or 4‐hexylphenylazosulfonate (Azo) (Brown et al., 2019). Their usage makes the sample preparation easier since no detergent removal is necessary, but it may reduce the amount of identified proteins when compared to other detergents, especially SDS (Schmudlach et al., 2016), and increase the cost of sample preparation.

Next, chaotropic agents such as urea or thiourea dissociate hydrogen bonds in the peptide backbone, then reducing agents, for example, dithiothreitol (DTT), 2‐mercaptoethanol, or tris‐2‐carboxyethylphosphine (TCEP) break the disulfide S‐S bonds destroying the protein's secondary and tertiary structure. Reduced disulfide bonds can be protected from bridge restoration and oxidation by alkylation with 2‐iodoacetamide or acrylamide. This protein unfolding is crucial for enzymes to reach their cleavage sites and digest polypeptide chains with the best efficiency possible (Wierenga et al., 2002). The addition of protease and/or phosphatase inhibitors during lysis, which inactivate endogenous enzymes responsible for proteolysis and dephosphorylation, as well as pH‐stabilizing agents may protect proteins from degradation and allow longer storage of samples before preparation and analysis.

Examples of commercial and self‐made lysis buffers used for protein extraction in sEV are summarized in Table 1. As shown, frequently used radio immuno precipitation assay (RIPA) buffers can vary greatly in their composition among various users. Even though a plethora of ingredients, lysis buffer recipes, and usage protocols are available, only limited information about their performance is available when sEV protein extraction and subsequent MS profiling are the objectives. An extensive comparison between various lysis buffers for sEV samples was published by Subedi et al. (2019). For example, it showed that up to 16% more proteins could be identified when the vesicles were lysed using Thermo Scientific™ RIPA buffer compared to other RIPA buffers. This may be due to the use of both ionic (SDS, SDC) and non‐ionic (NP‐40) detergents in RIPA, which allowed for solubilization and identification of numerous membrane proteins by this lysis method. Multiple studies indicate that the presence of detergents increases the number of identified proteins when compared to solutions containing only chaotropic agents or salts (Glatter et al., 2015; Neset et al., 2022). It should be noted, that sample preparation with detergent removal is longer and more expensive as compared to simple ‘in‐solution digestion’. Moreover, some studies suggest that certain groups of proteins are poorly soluble in RIPA, specifically the cytoskeletal and extracellular region proteins (Ngoka, 2008).

**TABLE 1:** Examples of lysis buffers used for extraction of sEV proteins; in the case of self‐made lysis buffers names/acronyms are given according to the cited references.

Name	Composition	References

Commercial buffers		
Cell Lysis Buffer *Cell Signaling Technology™* *no.cat.: #9803*	20 mM Tris–HCl, 150 mM NaCl, 1 mM Na_2_EDTA, 1 mM EGTA, 1% Triton, 2.5 mM Na_4_P_2_O_7_, 1 M beta‐glycerophosphate, 1 mM Na_3_VO_4_, 1µg/mL leupeptin, optionally: 1 mM PMSF	(Subedi et al., 2019)
RIPA *Thermo Scientific™* *no.cat.: 89900*	0.1% SDS, 1% NP‐40, 1% SDC, 25 mM Tris–HCl pH 7.6, 150 mM NaCl	(Cao et al., 2021; Le Gall et al., 2020; Subedi et al., 2019)
RIPA 10x *Cell Signaling Technology™* *no.cat.: #9806*	20 mM Tris–HCl pH 7.5, 150 mM NaCl, 1 mM Na_2_EDTA, 1 mM EGTA, 1% NP‐40, 1% SDC, 2.5 mM Na_4_P_2_O_7_, 1 mM beta‐glycerophosphate, 1 mM Na_3_VO_4_, 1 u*g/mL leupeptin, optionally: 1 mM PMSF	(Jung et al., 2020)
RIPA 10x *Merck Millipore™* *no.cat.: 20–188*	0.5 M Tris–HCl pH 7.4, 1.5 M NaCl, 2.5% deoxycholic acid, 10% NP‐40, 10 mM EDTA	(Yang et al., 2020)
RIPA 10x *Sigma‐Aldrich™* *no.cat.: R0278*	150 mM NaCl, 1.0% IGEPAL CA‐630, 0.5% SDC, 0.1% SDS, 50 mM Tris pH 8.0	(Soares Martins et al., 2018)

Self‐made lysis buffers		
RIPA	150 mM NaCl, 5 mM EDTA, 50 mM Tris–HCl, 0.5% NP‐40, 1% SDC, 1% Triton X‐100, 0.1% SDS at pH 7.4, 1x HALT protease and phosphatase inhibitor	(Skalnikova et al., 2019)
RIPA	50 mM Tris pH 6.8, 150 mM NaCl, 1 mM EDTA, 1% NP40	(Payton et al., 2021)
RIPA	150 mM NaCl, 1% NP‐40, 0.5% SDC, 0.1% SDS, 50 mM Tris pH 8.0	(Kw & Kierulf, 2015)
RIPA	50 mM Tris–HCl pH 7.2, 0.05% SDS	(Barberis et al., 2021)
exoLB	6% SDS, 0.2 M DTT, 200 mM Tris–HCl pH 7.6	(Pietrowska et al., 2017)
Tissue lysis buffer	4% SDS, 0.1 M DTT, 100 mM Tris–HCl pH 8.0	(Abramowicz et al., 2018; Gawin et al., 2018; Zebrowska et al., 2022)
SDT	4% SDS, 0.1 M DTT, 150 mM Tris–HCl pH 8.0	(Jiang et al., 2019; Ni et al., 2021)
Lysis Buffer II	25 mM Tris pH 7.5, 120 mM NaCl, 1% Triton X‐100, 1% PMSF, 1 mM NOV, 1 mM leupeptin	(Mutschelknaus et al., 2017)
–	4% SDS, 0.1 M DTT, 0.1 M Tris–HCl pH 8.0, 0.5% (w/v) polyethylene glycol 20000	(Ludwig et al., 2022)
–	4% SDS, 1% protease inhibitor cocktail	(Cao et al., 2021)
–	4% SDS, 0.1 M DTT, 50 mM triethylammonium bicarbonate	(An et al., 2017)
–	8 M urea, 1% protease inhibitor cocktail	(Ding et al., 2020; Wang et al., 2020)
–	30 mM Tris–HCl pH 8.0, 7 M Urea, 2 M Thiourea, 4% CHAPS	(Tsuno et al., 2018)

Most lysis buffers were originally designed for use with protein‐rich tissues and cells (Ngoka 2008; Winter & Steen, 2011). Thus, profiling some types of samples, like highly‐specialized immune cells (Geddes‐Mcalister & Gadjeva, 2019) or sEV proves challenging. As was mentioned before, the composition of a lysis buffer dramatically affects the efficacy of protein extraction (Ignatoski & Verderame, 1996). For this reason, one cannot underestimate the lysis buffer selection and the subsequent sample preparation optimization before MS analysis of sEV samples, where protein content is limited. Use of a lysis buffer containing ionic detergents, reducing agents, and protease inhibitors as a starting point for optimization can be recommended. Nevertheless, with properly selected buffers deep profiling of sEV proteins with MS is possible and yields reliable results.

### 4.2 sEV protein digestion

After sEV solubilization and protein denaturation, an appropriate protein digestion protocol must be implemented for the MS analysis to be carried out. Many such protocols have been published and comparisons as to their performance are available. Some examples of comparison studies are summarized in Table 2. Shortly, these methods include FASP, which utilizes filters (e.g. Microcon‐30 kDa) to stop proteins from eluting but allow contaminants and later peptides to be passed through. In the SP3 approach, magnetic beads are used to bind proteins and leave contaminants suspended in the solution. S‐Trap and in‐StageTip digestion (iST) methods capture proteins in the bed of a column, which allows the rinse‐out of contaminants and digestion to take place. Pressure‐assisted lysis and digestion (PCT) is a specific method that utilizes high pressure to accelerate the sample preparation, whereas Sample Preparation by Easy Extraction and Digestion (SPEED) achieves the same with a sample acidification‐neutralization approach. It is also possible to use short (∼1 cm) gel separation followed by an in‐gel digestion of the whole positively stained region to obtain the final peptide sample.

**TABLE 2:** Studies comparing different methods of protein digestion used for MS‐based proteomic profiling.

Methods	Sample type	Summarized comparison results	References
FASP, SP3, iST	Cell lysate	In the range of 10–20 u*g of starting material, iST allowed the highest number of IDs. Below that, SP3 and iST had similar results. The number of IDs for FASP was drastically reduced when <20 u*g of protein was digested. Below 20 u*g of starting material both SP3 and iST3 had high reproducibility.	(Sielaff et al., 2017)
FASP, S‐Trap, ISD	Cell lysate	S‐Trap based method outperformed ISD and FASP. ISD had least protein ID overlap and weakest correlation in technical replicates.	(Ludwig et al., 2018)
FASP, ISD, PCT	Cell lysate, FFPE tissue	ISD allowed for the highest amount of protein IDs for both cells and FFPE tissue. FASP allowed to identify the highest number of integral membrane proteins.	(Pirog et al., 2021)
FASP, S‐Trap, ISD, SPEED, iST	Cell lysate	Costs ranged from 1$ (ISD, SPEED, SP3) to 5$ (FASP), ∼10$ (S‐Trap), ∼20$ (iST), or ∼30$ (EasyPep) per sample. SDC‐based ISD method allowed for the highest number of IDs, followed closely by iST, FASP, and SP3. Several methods (SPEED, FASP, S‐Trap, and SP3) could benefit from further refinements, such as sequential digestion by trypsin and LysC.	(Varnavides et al., 2022)

Abbreviations: IDs, protein identifications; FASP, filter‐aided sample preparation; SP3, single‐pot solid‐phase‐enhanced sample preparation; iST, in‐StageTip digestion; ISD, in‐solution digestion; PCT, pressure‐assisted lysis and digestion; SPEED, sample preparation by easy extraction and digestion.

The number of identified proteins is highly dependent on the sample preparation protocol used. One of the most crucial initial parameters is the amount of protein available for digestion. It should be noted that the buffer used to solubilize sEV protein must be compatible with the chemicals used in protein concentration determination, such as bicinchoninic acid in the BCA assay. Usually, lysis buffers containing reducing agents (like DTT) are incompatible with such assays. Thus, the addition of a reducing agent must be performed at a later stage of sample preparation. In certain cases, a minimal amount of reducing agents may be tolerable (e.g. up to 5 mM of DTT in BCA assay (Thermo Scientific), although their concentrations are typically exceeded in lysis buffers except RIPA buffers. Whereas FASP is a method that yields very reliable results when tens of micrograms of protein are digested, some studies report poor reproducibility and the number of identified proteins at lower loadings when compared to other methods (Sielaff et al., 2017). This is exactly the case for sEV sample preparation, where the amount of protein is limited. Since the FASP method is comprised of multiple steps of washing using filters, this may lead to substantial sample loss. On the other hand, FASP yields a higher number of peptides originating from membrane‐bound proteins (Pirog et al., 2021). Although, it may seem that in‐solution digestion (ISD) methods would be an appealing choice for sEV preparation, these methods are incompatible with certain detergent‐based sEV lysis methods, for example, with high concentration SDS containing buffers. Other methods, like SP3, may be more expensive to utilize or have an upper limit to the amount of protein that can be captured and digested. On the other hand, in‐gel digestion requires a certain amount of sEV protein (e.g. 50 u*g) to be performed effectively (Wang et al., 2012). Thus, choosing a method of sample preparation is highly sample‐dependent and should be optimized beforehand.

It should be mentioned that sample preparation methods are validated on tissue or cell samples and much less information is available for small extracellular vesicles. It remains unanswered whether these results correspond to sEV sample preparation. The amount of protein, lipids and nucleic acids, which may or may not impact sample preparation efficiency, will be vastly different in cell or tissue samples when compared to sEV. The effect of such influence is hardly mentioned when sample preparation techniques are compared. Only the balance between protein‐detergent ratio has been previously mentioned in the literature (Wiśniewski, 2019). Moreover, in nearly all cases trypsin is used as the only digestion enzyme for sEV proteomic profiling. Yet, comparative studies suggest that a combination of other proteases may improve the number of identified proteins, even several fold (Dau et al., 2020). Enzymes such as chymotrypsin, pepsin or pancreatin have been already successfully used in sEV studies (Burkova et al., 2019; Chen et al., 2016; Hüttmann et al., 2024; Ogawa et al., 2021). As compared to cellular protein digestion, sEV may be also characterized by higher resistance to proteolytic enzyme action (Askenase, 2022). Another problem, like the case of naming the RIPA buffer, is the fact that researchers use the same blanket term of ‘in‐solution digestion’ to describe a plethora of different sample preparation protocols, that do not use any additional accessories, such as centrifugal filters etc. Thus in practice, comparing such techniques to each other becomes unreliable. Moreover, results obtained in different studies may not necessarily agree with each other, for example, S‐trap performance was lower than ISD in the study by Varnavides et al. (2022) but higher in the study by Ludwig et al. (2018). Taking all of this into account, selecting the proper method for protein digestion should be performed in advance of an experiment. This is especially the case for sEV samples where the amount of protein is limited. This limitation is both due to biological limitations and since part of the sample has to be used to characterize sEV according to the MISEV 2023 guidelines (Welsh et al., 2024). Unfortunately, to our knowledge no papers have been published that would directly compare sEV sample preparation methods for subsequent proteomics studies.

### 4.3 Sample complexity reduction

**FIGURE 3:**
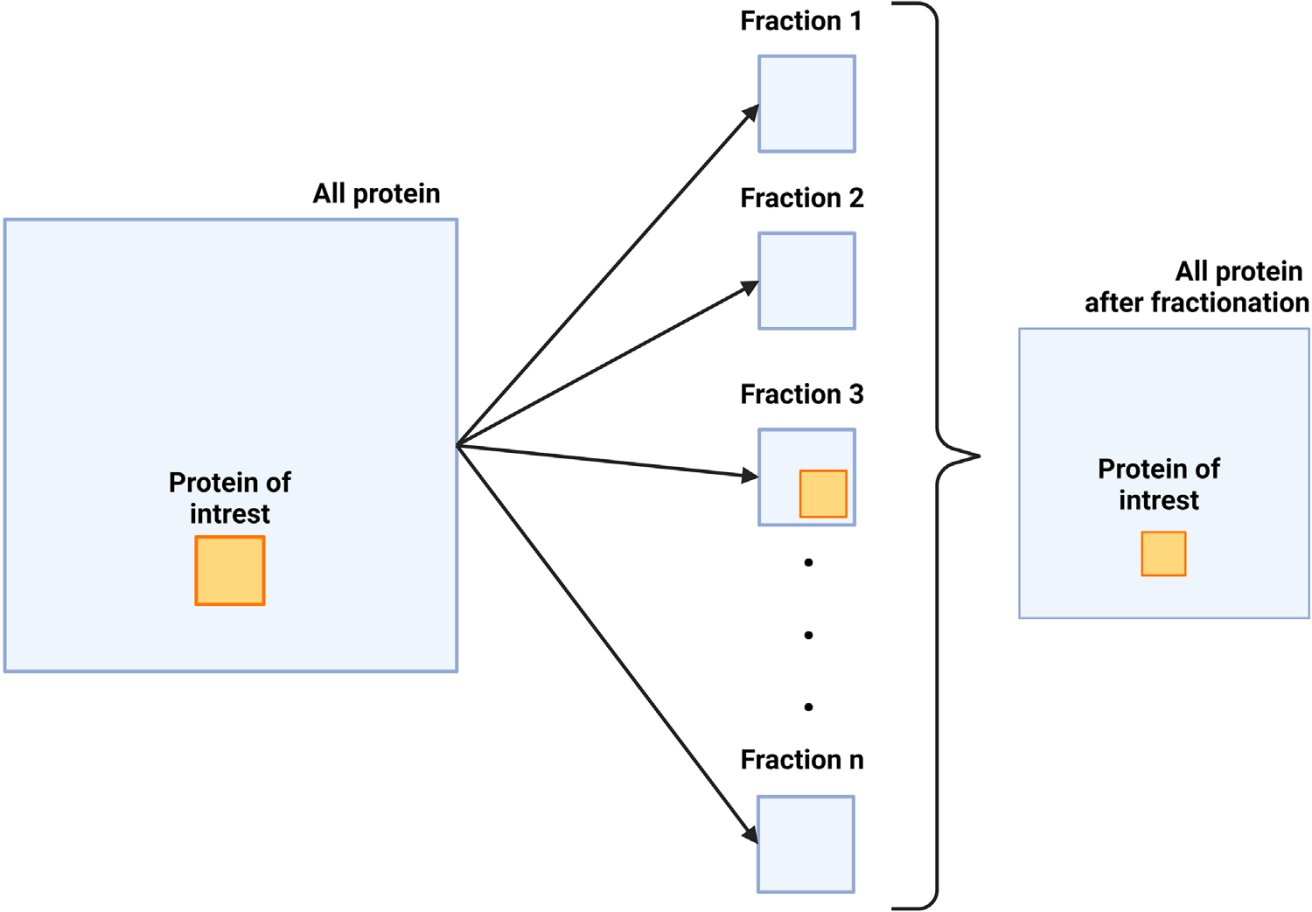
In a complex protein background, a selected protein of interest (POI) will constitute only a small fraction of all protein which poses a difficulty in its measurement using MS. Such sample can be divided into fractions, one of which will contain the POI in a more substantial amount aiding in its detection and quantification. On the other hand, due to losses during fractionation the total amount of protein is reduced, limiting the number of fractions that can be realistically obtained.

An important aspect of sample preparation is the sample complexity reduction (Figure 3). Due to the technical aspects of data‐dependent acquisition, which are described in more detail in the chapter ‘*Analysis type*’, the relative abundance of each protein in the sample should be roughly similar during measurement. The high disparity in selected protein abundance compared to others will lead to a poor number of protein identifications. Thus, to enrich a sample in the low abundant proteins, methods of complexity reduction have been developed. Moreover, during sample preparation proteins are digested into peptides increasing the complexity of the mixture—from hundreds or thousands of proteins to tens of thousands of peptides. Technical ability to identify so many unique molecules is limited and so complexity reduction may be necessary to allow acceptable proteome coverage. A proper method for sEV isolation may be considered the first step in this reduction. As compared to the complexity of biological fluids, sEV contain only a limited number of proteins and the broad range in their abundance may be reduced (Nigjeh et al., 2017).

Historically, one of the first methods to be utilized was protein separation by either 1D or 2D electrophoresis. Depending on the molecular weight and/or isoelectric point (pI) of each protein, they can be separated, excised from the gel, and used for digestion (Mears et al., 2004; Shevchenko et al., 2006). Based only on pI, in‐gel or in‐solution isoelectric focusing (IEF) could be utilized. This method is high time‐consuming, thus has largely fallen out of favor. Ion‐exchange chromatography (IEX) has been used to achieve separation based on the pI of the peptides with their elution using solutions of stepwise changing pH or salt concentration. StageTip fractionation using strong anion exchange (SAX) of peptides obtained from tissue samples has been used to increase the number of identified proteins without any special equipment or procedures (Wiśniewski et al., 2009). On the other hand, high‐pH reversed‐phase fractionation, where acetonitrile gradient is used to elute peptides, allows separation without subsequent desalting (Yang et al., 2012). Even if no strategies for ‘offline’ sample fractionation are used, standard MS‐based proteomics analysis consists of separating peptides using reversed‐phase liquid chromatography (RPLC). Depending on the hydrophobicity of the peptides, they can be characterized by differing retention times. This dimension of separation may be sufficient for some types of analyses and can be characterized by limited dead volume. Additional separation dimension can be achieved by adding another ‘online’ column used for peptide fractionation, such as an ion‐exchange or hydrophilic‐interaction (HILIC) column. The latter was successfully used to increase the number of identified proteins up to 34% in tissue samples (Roca et al., 2021).

Again, very limited information for sEV fractionation performance is available as compared to cells or tissues. Based on our unpublished results, SAX separation into two fractions yields better results for serum‐derived sEV than no fractionation. On the other hand, using the original separation into six fractions as reported by Wiśniewski et al. (2009) yielded worse results than no ‘offline’ complexity reduction at all (Wiśniewski et al., 2009). It may be the case, that the amount of available material for sEV SAX fractionation is so limited that it is rarely performed. Especially for ‘offline’ fractionation, the losses that occur may be significant enough to dissuade from using this technique as a part of standard sample preparation methodology. Nonetheless, reverse‐phase fractionation has been successfully used in some sEV studies (Fujita et al., 2017; Li et al., 2021). MS analyses other than DDA may be used to at least partially alleviate the problem of sample complexity reduction, such as these described in chapter ‘*Analysis type*’. On the other hand, methods that require prior knowledge about the sample will need to be analysed using DDA anyway to obtain a spectral library or this library will have to be generated completely in silico.

## 5 MS ANALYSIS OF SEV

Several analytical techniques can be used for the analysis of proteins extracted from sEV. Some of the commonly used techniques include Western Blot, ELISA, protein microarrays, gel electrophoresis, or fluorescence microscopy. These techniques can be used in combination to provide comprehensive information about the protein content of sEV, including their composition, abundance, and post‐translational modifications, but the method of choice for sEV proteomics became mass spectrometry. This is due to many factors, such as high sensitivity, high throughput, compatibility with small sample sizes, etc. There are several analytical approaches involving MS which may be used for successful analysis of proteins extracted from sEV which will be discussed in detail in this chapter.

### 5.1 Advancements in MS technologies

MS‐based proteomic analyses are constantly developed and improved by advancements in analyzers and related technologies used in mass spectrometers themselves. These developments allow to achieve higher number of protein identifications, which are usually tied to the spectrometer's scanning speed. For example, the first Orbitrap mass spectrometer—Thermo Scientific's LTQ Orbitrap achieved up to 5 Hz of scanning speed (Eliuk & Makarov, 2015). Latest instrument of a similar type—Thermo Scientific's Orbitrap Astral archives up to 200 Hz with single ion detection sensitivity (Heil et al., 2023). Thus, by the development of the instrumentation alone, the theoretical number of identifications per second has increased severalfold over the years. In studies of sEV, Orbitrap Astral has been used in research of extracellular vesicle‐enriched plasma where 5163 proteins were identified using a 60‐minute gradient. Using the same sample enrichment protocol, a different research group was able to achieve up to 4163 protein identifications with a 110‐minute analysis on an older Orbitrap Eclipse MS system (Heil et al., 2023; Wu et al., 2024). Using the newest Orbitrap Astral, a nearly two times shorter analysis still allowed to obtain a slightly higher number of protein identifications. Moreover, what is especially important in case of sEV, which can be characterized by a limited amount of protein as compared to cells or tissues, the sensitivity of MS systems has significantly improved over time. Some of the first reports for the most recently announced Thermo Scientific's Stellar MS system, a linear ion trap (LIT), show acceptable results even at the 1 ng level due to the spectrometer's high sensitivity (Plubell et al., 2024). Historically, targeted analyses (i.e., MRM/PRM) were limited in the number of proteins that could be monitored during a single run. With the newest Stellar MS, it is now possible to analyze thousands of peptides with acceptable peak coverage (Remes et al., 2024). This system is yet to be validated in biological studies of extracellular vesicles, but it remains a promising tool for ultra‐sensitive quantitative measurements of sEV biomarkers. The ability to measure ions in the form of packets released from a trap is an advancement that increases the sensitivity of the instrument. When combined with a TOF instrument, packet analysis allows to boost the duty cycle of the whole MS system. Such an approach, used by SCIEX in the ZenoTOF spectrometer, has been successfully employed to identify 5179 proteins, where an analysis without this ‘trapping and pulsing’ reached only 2743 identifications (Wang et al., 2022). Again, as with the Stellar MS, this system is yet to be validated in studies of sEV.

### 5.2 Ion mobility

A separate advancement that has recently gained a lot of interest is the analysis of ion mobility in sEV proteomics studies. This group of emerging techniques propose a solution to the problem of complexity reduction, where separation of ions is based on their mobility. Ion mobility spectrometry (IMS) was developed in the 1960s but only recently it has been successfully miniaturized and used in bottom‐up proteomics research (McDaniel et al., 2004; Meier et al., 2018). The ability to distinguish between molecules of the same *m*/*z* based on additional parameters, such as ion mobility, adds another separation dimension to the analysis. Moreover, IMS is an ‘online’ technique of separation that does not require special sample preparation procedures, as was the case with the methods described in the chapter ‘Sample *Complexity reduction*’.

Some of the first devices used for IMS were drift tubes, which would separate ions during their travel in a uniform electric field with an opposing drift gas flow. These devices have not been used in the studies of macromolecules due to technical limitations in their resolving power. More modern techniques have been built on the idea and have been successfully employed in proteomic studies. First, trapped ion mobility spectrometry (TIMS) is a technique that traps ions using gradient electromagnetic force and drift gas flow in an opposing direction. In a dual‐TIMS equipped device, ions can be trapped in the first TIMS, and allowed as packets into the second TIMS where they are sequentially eluted based on their mobility. This approach, called Parallel Accumulation‐Serial Fragmentation (PASEF) utilizes nearly all available ions, thus no potential signal is lost during the analysis. TIMS has been coupled to time‐of‐flight (TOF) detector in the Bruker timsTOF Pro and Pro2, TIMS SCP, TIMS TOF Flex MALDI, TIMS TOF ULTRA and TIMS TOF HT spectrometers (Bruker Daltonics; Bruker Daltonics). High‐field asymmetric‐waveform ion mobility spectrometry (FAIMS) is another technique utilizing ion mobility for their separation. Here, an asymmetric electric field changing at a low and high frequency with drift gas flow is used to allow the passage of ions with selected mobility through the device. This method cannot be described as an ion mobility, since it functions to filter out certain ions, thus some of the potential signal is lost during the analysis. On the other hand, interference caused by a high abundance of some peptides becomes limited and the relative intensity of low abundant molecules is boosted. This is also the case for chemical noise caused by the presence of singly charged ions. ThermoScientific's FAIMS Pro Duo interface can be coupled to mass spectrometers such as Exploris480 (Bekker‐Jensen et al., 2020). Other novel examples of ion mobility separation methods include Waters’ Cyclic IMS and MOBILion Systems’ SLIM. In the case of cyclic IMS, ion packets are injected into a circular ion guide where they can be spun for an extended period of time, allowing for their separation (Eldrid et al., 2019). SLIM (i.e. structures for lossless ion manipulation) separates ions during their migration in a long path up to 13 m, which can be thought of as a modern approach to the drift tube concept (May et al., 2021).

Due to the novelty of this separation method in the study of proteins, its potential is yet to be verified. First studies show promising results, for example, PASEF was first successfully employed to aid in the identification of more than 6000 protein groups in a 200 ng single‐run for HeLa standard digest (Meier et al., 2018). Specifically, in case of small extracellular vesicles, one study used the PASEF method to identify 3466 unique protein groups in a triplicate run of cell‐derived sEV (Buck et al., 2022). Serum‐derived vesicles were characterized using this technique with 915 protein identifications in a study comparing samples from healthy donors and patients suffering from dermatomyositis or polymyositis (Meng et al., 2023). An optimization study in the field of proteome research of cerebrospinal fluid‐derived sEV achieved around 743 identifications (Kangas et al., 2023). FAIMS interface was used in the analysis of serum‐derived sEV from colorectal cancer patients. Here, its use with two compensation voltages allowed to increase the number of identifications by 30%, up to 559 protein groups, when compared to the non‐FAIMS analysis (Montero‐Calle et al., 2023). The number of studies showing the effectiveness of novel IMS techniques (e.g., SLIM or cyclic IMS) in the characterization of sEV is very limited. Depending on the amount of starting material, method of sEV isolation, and sample preparation, the number of identified proteins is highly variable. Taking that into account, methodological studies that directly compare IMS and non‐IMS proteomic analyses of sEV are needed.

### 5.3 MS analysis type

Understanding the mechanism of the MS analysis type itself is very important since it dictates the sample preparation and separation procedures that precede it. One of the most widely used techniques of MS analysis in the case of sEV proteome profiling is the data‐dependent acquisition. Here, a set number of precursors identified in MS^1^ mode are chosen for fragmentation and the resulting fragments are detected in the MS^2^ mode. The most crucial aspect of choosing such precursors is their peak area, representing their abundance in the sample. Since the fragmentation is performed using precursors chosen on the fly, this approach is described as data‐driven or data‐dependent. Although it works remarkably well in most analyses, certain limitations of the technique should be noted. First, samples that contain a substantial amount of some proteins and a relatively low abundance of others, may be difficult to analyze using DDA. This is the case for sEV samples originating from serum or plasma, where albumin and immunoglobulins are highly abundant and make up to ∼75% of total serum protein weight (Jaros et al., 2013). The same problem may be observed in samples from urine where albumin and uromodulin may be considered contaminants. These highly abundant proteins will be often chosen for fragmentation producing redundant MS^2^ spectra and thus reducing the number of available scans that could be used to identify low‐abundant, but biologically important proteins (Skoczylas et al., 2024). The need to reduce the amount of contaminating proteins has been already recognized and certain solutions for specific protein depletion have been implemented (Bellei et al., 2011; Björhall et al., 2005; Borberg et al., 2021; Mrozinski et al., 2008; Zougman et al., 2020). Regardless of the sample type, fractionation methods can be utilized, but require special sample preparation and much longer analyses. Specifically, in the case of sEV the number of contaminants will vary based on the isolation technique used, though any technique may yield vesicles that are coated with proteins, that is, vesicles with a protein corona that could not have been removed during isolation. DDA is a method that can be regarded as method of lower reproducibility compared to DIA, since variations in the selection of precursors for fragmentation may introduce variability in MS^2^ spectra acquisition (Li et al., 2021). This will in turn influence the number and type of proteins identified in an experiment. Certain additional standard method improvements, such as dynamic exclusion of precursors or matching between runs can help in yielding higher reproducibility. Nonetheless, due to the ease of implementation and no need for any a priori knowledge about the sample, DDA has been used extensively in sEV research  (Kugeratski et al., 2021; Pedersen et al., 2022; Pietrowska et al., 2017; Zebrowska et al., 2022; Zhu et al., 2022).

Another technique, data‐independent acquisition (DIA), is a method that largely addresses the shortcomings of the DDA approach. Whereas in DDA, MS^2^ spectra are generated for chosen and well‐defined precursors, in DIA many precursor ions that fall into a certain isolation window are fragmented at the same time. Later, bioinformatical tools and methods are used to extract pseudo‐MS^2^ spectra from the raw data and an a priori obtained spectral library (Wang et al., 2022). Here, all the possible information to be gathered during the analysis may be collected and the burden of understanding the results is carried over to the spectral library preparation and data analysis. Only the representative, often pooled sample used to generate the spectral library must be meticulously prepared, fractionated, and analysed in DDA mode. Alternatively, complete in silico methods of spectral library preparation may be used, but their validity and accuracy are yet to be verified (Gessulat et al., 2019). Samples that make up the actual experiment do not need to be fractionated, although one study suggests that division into 6 pI‐based fractions and subsequent DIA analysis can increase the number of identified protein groups by around 35% as compared to non‐fractionated samples (Cho et al., 2020). With a well‐prepared spectral library, all the pseudo‐MS^2^ spectra can be assigned to peptides, thus allowing protein identification. This process is not susceptible to minor changes in the MS^1^ signal intensity, which drives the fragmentation process, thus DIA can be regarded as a highly reproducible method. In the end, all the described characteristics of the DIA analysis make it uniquely suited to sEV proteomics research. Samples of sEV are usually limited in the amount of protein available, so requiring no fractionation to achieve high proteome coverage is very beneficial. Moreover, the problem of highly abundant proteins is not strictly applicable to DIA (as here no redundant MS^2^ spectra are obtained, which may be the case in DDA) and no additional contaminating protein removal steps must be taken. On the other hand, even when DIA is utilized, abundant proteins can limit the amount of sample that can be loaded onto the column and may cause signal suppression at the source. With this set of advantages and disadvantages of DIA, a number of studies utilizing this analytical technique has already been published for sEV (Ding et al., 2024; Lattmann et al., 2024; Tian et al., 2024).

Both DDA and DIA are analysis modes used in exploratory studies. Their implementation is made in a way that allows the identification of all or nearly all of the proteins in the sample. Since no real measurement instrument, such as a mass spectrometer is infinitely sensitive, a need to implement more targeted approaches is present. This can be achieved by limiting the number of scans per unit time in multiple‐ or parallel‐reaction monitoring (MRM/PRM) modes. These modes are used to measure the signal from parent ions (i.e., peptides) and their corresponding fragments either sequentially (in MRM) or for all fragments at the same time (in PRM). Again, a priori knowledge is required to select the peptides and their fragment ions that will be monitored, either during the whole analysis (in an unscheduled analysis) or during certain retention time windows (in a scheduled analysis). Targeted analyses are most useful after an exploratory proteomic study is completed and selected proteins are chosen for further validation. MRM/PRM modes are characterized by the highest statistical confidence but are limited in scope to only selected proteins as compared to DDA/DIA. It should be noted that novel mass spectrometers, such as the Thermo Stellar, can still measure thousands of peptides (up to 8000) during one run with an acceptable peak coverage (Remes et al., 2024). These new technical developments thus blur the line between targeted and untargeted methods. In MRM/PRM, specially made stable isotope‐labeled standard peptides can be used to measure the protein abundance in an absolute quantitative manner. This approach of exploratory analysis, followed by targeted analysis has been used in many proteomics studies of small extracellular vesicles  (Ni et al., 2021; Soloveva et al., 2023; Yu et al., 2023).

### 5.4 Other sEV MS‐based approaches

To provide a broad overview of MS‐based sEV profiling, some specialized forms of sEV analyses should be briefly mentioned. Although sample preparation and data analysis differ from LC‐ESI‐MS/MS‐based techniques, their impact on sEV research cannot be overstated. These include analyses with different ionization methods (e.g. MALDI) of intact proteins (i.e. the ‘top‐down’ approach) as well as the characterization of post‐translational modifications of sEV proteins.

Compared to electrospray ionization (ESI) used widely in mass spectrometry proteomics research, matrix‐assisted laser desorption ionization (MALDI) may be used as well. Due to its ease of use and robustness, it provides an attractive choice in high‐throughput screening of certain proteins (Duncan et al., 2016). Here it is possible to ionize molecules with a substantially higher mass and thus mass spectra with a wider *m*/*z* range can be obtained, even up to tens of kDa. Consequently, acquiring such spectra for whole sEV proteins becomes possible and no special sample preparation steps (e.g. digestion) must be taken. A simple comparison of MS profiles between the samples may yield insight into the differences in experimental conditions, without any need for difficult data analysis. This ‘fingerprinting’ approach in sEV research has been used successfully and allowed, for example, to distinguish samples of sEV from mice with and without subcutaneous melanoma (Zhu et al., 2019). In another study, sEV from patients with osteosarcoma could be used to differentiate between patients that developed a lung metastasis and patients that did not (Han et al., 2021). On the other hand, extracting enough information to determine the presence of specific proteins with acceptable proteome coverage is not achievable using this technique. Additional and more targeted methods must be used if significant differences in spectra have been identified. If this specific route of analysis is to be performed, it is important to notice that the operating conditions can greatly affect the outcome. For example, the choice of an inappropriate matrix and its concentration, too low or too high a number of laser shots may impact the result and in some cases lead to no signal detected at all (Rajavel et al., 2023; Yu et al., 2021). Thus, appropriate optimization of experimental conditions should be the first step in such analyses.

Another approach to sEV protein analysis is the determination of their post‐translational modifications (PTMs). Since vesicles carry proteins that can be representative for their cell of origin, the pattern of PTMs may hold crucial information about the state of such cells. As an example, in cells PTMs take part in de‐/activating protein signalling pathways by phosphorylation, mediating cellular recognition by glycosylation or marking proteins for subsequent degradation by ubiquitination, etc. An important aspect of sEV‐specific PTMs is vesicle formation and biogenesis, which can be regulated by the pattern of their protein PTMs  (Carnino et al., 2020; Moreno‐Gonzalo et al., 2018; Romancino et al., 2018). Moreover, the distribution of sEV in different tissues may be PTM‐dependent (Royo et al., 2019). MS‐based analysis of sEV protein PTMs can provide an abundance of information that exceeds the possibilities of standard proteomics analyses (Gonzales et al., 2009; Rosa‐Fernandes et al., 2017; Smolarz et al., 2022). Since the role of PTMs specifically in sEV is not yet fully explored, more studies are needed to understand their function.

## 6 MS DATA ANALYSIS

### 6.1 Raw data analysis

The final step in proteomic profiling using mass spectrometry can be broadly defined as ‘data analysis’. This term refers to the process of extracting information about protein abundance from raw MS data. It should be noted that this task is not trivial, since proteins in each sample have been enzymatically digested into their constituent peptides. Analysis software must reconstruct which peptides are present in the sample together with their abundance, then use this algorithm to quantify the total protein abundance. All of this information has to be extracted from the raw data, which can be visualized as a three‐dimensional space with axes representing retention time, mass‐to‐charge ratio, and signal intensity. If IMS is used, this adds another dimension of separation. In either case, data is annotated with information gathered from MS^2^ analysis, where ions detected in MS^1^ mode are fragmented.

Usually, raw data is recalculated into protein abundance using ready‐made software—either commercial or freely available for the general use of the scientific community. It is also possible to use self‐made programs and software packages, but this approach demands advanced programming skills and knowledge of bioinformatics. Currently, some of the best‐established solutions for DDA data analysis include the commercial ThermoScientific's Proteome Discoverer (Proteome Discoverer Software) (PD) and freeware MaxQuant (Cox & Mann, 2008) (MQ), both available since at least 2008  (Cox & Mann, 2008; Thermo Fisher Scientific Inc). A comparison of the performance of the above‐mentioned software revealed that PD gave better quantification yield, dynamic range, and reproducibility than MQ, although MQ generally reached slightly higher specificity, accuracy, and precision values. For low‐abundant proteins, PD could reach even up to two‐fold higher quantification rate than MQ (Palomba et al., 2021). In another study, MQ performed better in terms of accuracy and precision, whereas PD—in terms of quantifiable low abundance proteome coverage (Zhao et al., 2020). A newer candidate in terms of data analysis is the Fragpipe (FP) software package utilizing the MSFragger engine—when compared against PD it identified a similar number of proteins (PD – 9178 vs. FP – 9656) in a vastly shorter amount of time (PD – 673  min vs. FP – 5.4  min) (Kong et al., 2017). Other solutions for data analysis are available, but there is very limited information as to their performance. Notable examples include PEAKS Studio (Zhang et al., 2012), OpenMS/TOPP (Reinert & Kohlbacher, 2010), or MScan (Malinowska et al., 2012).

Analysis of DIA data requires a completely different algorithm, as the information contained in the MS^2^ scans differs between DDA and DIA approaches. Some DDA data analysis programs support DIA—this includes ProteomeDiscoverer, MaxQuant or Fragpipe. Nonetheless, there are also DIA specific programs which are often used instead, such as DIA‐NN, EncyclopeDIA, OpenSWATH, or Spectronaut (Baker et al., 2024; Demichev et al., 2020; Röst et al., 2014; Searle et al., 2018). Multiple studies have already been published which benchmarked their performance (Fröhlich et al., 2022; Lou et al., 2023; Zhang et al., 2023). In most, DIA‐NN is evaluated to have better proteome coverage, quantification precision and accuracy than other programs. In a study by Zhang et al. this difference in some conditions was more than two‐fold in the number of identified proteins (DIA‐NN – 2295 vs. EncylopeDIA – 928) (Zhang et al., 2023). Quantification reproducibility of DIA‐NN was also slightly higher than the reproducibility achieved by other tools (median CVs of DIA‐NN 4.9‐11.8% vs. Spectronaut 6.1%–20.2%) (Lou et al., 2023). It should be noted that although general conclusions can be made about DIA data analysis programs, they are often updated, and improved, may or may not use spectral libraries, etc. sEV prove especially difficult in their analysis—the dynamic range of MS systems is limited and sEV often contain a lot of highly abundant proteins. Thus, a confident analysis of biologically relevant low‐abundant proteins is difficult to discern out of noise. Verifying which of these solutions will provide the optimal results should be included in the complete proteomic workflow.

Users must precisely define their goals in data analysis depending on the task at hand. In some cases, better identification coverage of the proteome can be advantageous, that is, when qualitative analysis is to follow. But it may as well be disadvantageous, when quantitative data are to be compared between experimental groups and numerous less precise identifications could impact multiple testing correction penalties. Choosing the best workflow does not add any additional cost, while significantly boosting the performance of the analysis. As such, optimizing and standardizing its use will yield the best results possible.

### 6.2 Data normalization approaches

Regardless of the method for raw data recalculation, the resulting matrix of abundance values for each protein in each sample must be normalized. Small variation is the amount of sample used in the MS analysis will have an impact on the level of protein abundance in a sample. Moreover, this impact may not necessarily be equal for all peptides (and consequently proteins) due to their differing ionization efficiency. Thus, normalization approaches are highly variable, considering only some or nearly all of the factors that make it necessary to normalize the data in the first place (Dubois et al., 2022; Välikangas et al., 2018). The easiest normalization approaches perform only mathematical operations on the available data. A notable example of such technique is the median normalization. It does not consider differences in the ionization efficiency of all peptides. On the other hand, sophisticated normalization can be performed with the aid of the data analysis programs, for example, MaxLFQ algorithm implemented in MaxQuant (Cox et al., 2014). It should be noted that removal or inclusion of certain contaminants, such as keratins originating from the sample preparation, will impact the normalized values and may necessitate additional normalization at a later stage of the data analysis. These normalization methods are used ubiquitously in label‐free proteomics since they require no additional cost or special sample preparation, but their performance is limited (Stepath et al., 2020).

Normalization may also be achieved with the use of appropriate internal standards. Ideally, internal standards in the form of stable isotope‐labeled peptides from all possible proteins would be spiked into the sample (Brzhozovskiy et al., 2022; Gillette & Carr, 2013; Picotti & Aebersold, 2012). This way, they would serve as a perfect analogue with the same ionization characteristics as the native form and could be used to determine the concentration of all proteins in an absolute quantitative manner. The ability to obtain and detect all such standards remains elusive and this can be realistically only achieved when targeted MS analyses are performed for selected proteins. The use of stable isotope‐labeled peptides may still be incorporated in exploratory studies in an abridged form—mostly to correct for loading differences during sample injection. Another, yet similar approach is to use intact proteins from other species than those from which the sample is prepared (Uszkoreit et al., 2022). In this way, digestion and loading differences can be assessed. These proteins must be chosen carefully—they have to produce peptides with limited similarity to the ones found natively in the sample. Moreover, they must be available, chemically well‐defined (i.e., pure), and stable over long‐term storage. Lastly, labeling of all peptides originating from a chosen sample can be used to determine the protein abundance of multiple samples during a single MS analysis run (Thompson et al., 2003). This approach, called tandem mass tag (TMT) labeling, eliminates run‐to‐run variability, peptide ionization differences and allows to quantify proteins with higher confidence. On the other hand, TMT is not perfect as the number of samples that can be analyzed at once is limited and highly costly. Moreover, quantification confidence may become eroded when problems such as ‘ratio compression’ are considered. This phenomenon causes a systemic underestimation of protein abundances since in complex samples coeluting peptides found within the isolation window may introduce interference (Karp et al., 2010; Madern et al., 2024; Savitski et al., 2013). Nonetheless, as an example, TMT was utilized in a study of sEV from healthy donors and triple‐negative breast cancer patients (Li et al., 2021). This technique could also aid in detecting the differences in sEV from healthy donors and ovarian cancer patients (Zhang et al., 2019). Spike‐in methods described above require manual intervention in the makeup of the sample. Alternatively, stable isotope‐labelled (SIL) amino acids can be used as a part of a cell culture medium or feedstock for animals allowing for their growth with metabolic incorporation of such amino acids (Krüger et al., 2008; Ong et al., 2002). This Stable Isotope Labelling by Amino Acids in Cell Culture (SILAC) technique ends with a MS measurement of a pool of SILAC and non‐SILAC samples in a single run. Again, as was the case with TMT (Tandem Mass Tag) labeling, this eliminates run‐to‐run variation and inconsistencies due to ionization differences. SILAC is also a method that can be characterized by higher cost and is limited in the number of experimental conditions. It also means that the complexity of the sample is multiplied, thus the amount of data that is gathered in unit time should be adjusted accordingly when compared to label‐free approaches. This is especially important since, for example, in DDA 2‐plex SILAC workflow theoretically 50% of precursors will be redundant as they constitute a SILAC isotopic pair (Pino et al., 2021; Smith et al., 2022). In sEV research, SILAC was successfully employed in quantifying the differences in the proteome of sEV from TGFBR2 proficient and deficient colorectal cancer cells (Fricke et al., 2019). As another example, this method was also used for MS‐based analysis of normal and lung cancer‐derived sEV (Clark et al., 2016).

Appropriate care should be given to minimize run‐to‐run variance and provide results of high quality which may be realistically achieved with isotope‐labelled samples if the number of analyses is relatively small. It is standard practice to use ready‐made and commercially available cell digests (e.g. HeLa cell digest) in benchmarking the performance of new workflows or systems. We recommend that sEV benchmarking be performed at the same time, since cell or tissue profiling can differ substantially from sEV profiling. This can be achieved using commercially available sEV standards to aid in the repeatability of the whole optimization process (Abcam; Creative Biolabs; Immunostep Biotech). Here, sEV from different sources should be investigated separately.

Specifically for vesicles, normalization may also take the form of quantifying and equalizing the number of particles that are used for the digestion in the first place. A multitude of methods for sEV quantification exist, one of which is the Nanoparticle Tracking Analysis (NTA) (Koritzinsky et al., 2017). It is often used in sEV research as part of conforming to the MISEV guidelines—information about the size distribution of particles is one of its requirements. Flow cytometry or transmission electron microscopy methods may also be used to achieve the same goal. If one method with high reproducibility is used for all samples, this may positively impact the variance of signal intensity at later stages. Nonetheless, the initial normalization based on the number of particles does not guarantee that the subsequent steps of sample preparation and MS analysis will be carried out equally for all. Thus, other normalization approaches may need to be used in parallel, regardless of the initial normalization to the number of particles.

In the end, an appropriate method of normalization has to be chosen for each experiment. If the number of samples is low and it is possible to use isotope‐labelled internal standards, their advantages should be exploited. This has been the case in multiple studies on sEV (Cheruiyot et al., 2017; Fricke et al., 2019; Huang et al., 2022). Whereas in case of high throughput experiments, the best mathematical normalization may be used since no labelling methods remain viable.

### 6.3 Contaminants in further data analysis

Part of the data analysis process must deal with contaminant removal. Even if best practices are followed during sample preparation, contaminants are expected in the resulting list of identified proteins. These can originate from the sample preparation itself (e.g. keratins) or due to the sEV isolation technique (e.g. lipoproteins) and due to their origin (e.g. serum albumin, FBS proteins) (Keller et al., 2008). Non‐protein contaminants that cause ion suppression or that obscure MS spectra cannot be accounted for at the stage of the data analysis and must be avoided in the first place. For protein contamination, if no steps are taken to remove them from the data, their presence will lead to false identifications that do not come about from biological, but only technical differences. The normalization of the data before this removal may also be hindered. Specifically, in the case of DIA proteomics analyses, the process of generating pseudo‐MS^2^ spectra and peak picking of fragment ions may be especially difficult if no care is given to removing the contaminants (Frankenfield et al., 2022).

There are lists of general contaminants, such as the common Repository of Adventitious Proteins—cRAP (which has not been recently updated) or a newer list prepared by Frankenfield et al. (2022), crap (nd). On the other hand, no standard list of protein contaminants specific for sEV samples has been published and widely accepted, thus every data analysis program takes its approach to deal with this problem. For example, general contaminants are marked by default by MaxQuant, but in ProteomeDiscoverer this has to be manually called for in the consensus workflow. As an effect of the lack of standardization in this regard, certain proteins which may be considered contaminants have been documented extensively in databases that collect and report sEV‐specific proteins. For example, albumin is reported as the top 25^th^ protein out of top 100 in the Vesiclepedia database (Chitti et al., 2024). It may be argued, that albumin may stick to the surface of vesicles creating the protein corona, and should be considered part of the sEV. On the contrary, general contamination lists include it as a known contaminant and there are no studies which would suggest that albumin could be considered part of the core sEV proteome. It remains unanswered, which proteins that are reported as sEV‐specific are in actuality just contaminants from the sample matrix, that is, serum, urine, etc. It may be advantageous to propose and use standard lists of sEV contaminants to differentiate between the core vesicle and its surroundings. Another approach may be to supply data entries with information about the localization of the protein in the lumen, membrane or corona (proximity) of the vesicle if such information is available. On the other hand, the existence of such data repositories which contain information about specific sEV proteins can serve as an additional tool in the data analysis process. These repositories include already mentioned Vesiclepedia, as well as ExoCarta, Urinary Exosome Protein Database, and Exosome Gene Ontology Annotation Initiative (Exosome Gene Ontology Annotation Initiative | European Bioinformatics Institute; Keerthikumar et al., 2016; Urinary Exosome Protein Database). With multiple researchers supplying high‐quality data it is possible to establish a reasonable ‘core proteome’ of sEV. A comparison of own results with such ‘core proteome’ may reveal insights into specific signalling pathway up‐ or downregulation.

## 7 SEV PROTEOMICS LIMITATIONS

All of the details that have been described above have a distinct purpose to provide reliable, repeatable, and accurate information about the sEV proteome. sEV proteomic profiles are promising to become prognostic factors in a variety of diseases in the form of a ‘liquid biopsy’ (Li et al., 2021; Yu et al., 2022; Zhou et al., 2020). Several recent studies have been focused on providing such profiles to show that sEV can serve as a surrogate of a donor cell or tissue proteome  (Braun et al., 2020; Hoshino et al., 2020; Hurwitz et al., 2016; Shen et al., 2023; Synadaki et al., 2019; Zheng et al., 2020). This is because sEV can directly reflect the proteome of cells that have released them (Garcia‐Martin et al., 2022). Nonetheless, several challenges and limitations still stand in the way of using MS‐based sEV proteomics in diagnostic procedures and in using such data to explore sEV‐mediated cellular communication.

sEV isolation methods can differ substantially and there is a multitude of methods to obtain an sEV population for subsequent analysis. Even if proteomic profiling is reliably performed, the results obtained for sEV isolated with differing techniques will be incomparable and inconsistent with each other (Jimenez et al., 2023; Torres et al., 2024). Using a combination of isolation methods may also be used, which has been shown to give the highest number of protein identifications when compared to non‐combined isolation methods, even at the cost of lower protein yield (Torres et al., 2024). Even if an isolation method has been used in the discovery of biomarkers, implementing changes or its simplification for diagnostics may provide unexpected results.

Another major hurdle is the fact that sEV do not natively contain a universal internal standard (IS) that could be used to normalize the obtained data. For cells or tissues, several IS had been proposed and used in proteomics studies. Examples include glyceraldehyde‐3‐phosphate dehydrogenase (GADPH), β‐actin, β‐tubulin, DJ‐1 (PARK7) and others (Li & Shen, 2013; Wiśniewski & Mann, 2016; Wu et al., 2012). In vesicles their presence does not necessarily correspond to the expression of proteins for the cell of origin. Thus, the number of vesicles released per cell, the amount of protein packed into each vesicle and a multitude of other factors may influence the results, without a straightforward ability to normalize the data. It should be noted that an sEV population is also highly heterogenic—certain vesicles are released by the cells of interest, but others may originate from unrelated cells or tissues. This is the case in serum or urine sEV proteomics. Although sEV originating from the organ that is of interest should be present in such fluids, most vesicles may instead originate from fibroblasts making up the blood vessels and unrelated tissues. One way to combat this shortcoming is the use of immunocapture isolation techniques, but this is tied to a significant loss in the amount of sEV protein for subsequent analysis. Making the distinction between reliable and unreliable data even harder, if the studied organism is under stress or the environmental conditions change, the proteomic composition of sEV will be severely impacted (Beninson & Fleshner, 2014; Harmati et al., 2019; Smolarz et al., 2022). In studies of the sEV proteome, biomarkers specific for a chosen disease are often under investigation. Since a selected disease poses stress upon the organism, distinguishing between disease‐specific markers and stress‐related proteins remains a challenge. Any change in the homeostasis of an organism will impact the result, without regard to the specific disease in mind. In the end, proteomic profiling of sEV is also complicated by the presence of biological contaminants, for example, serum albumin. MS‐based methods are not suited to differentiate between transmembrane or lumen protein of a vesicle and corona or co‐eluting protein present in the sample unless specific isolation methods are used. Unlike other types of material, sEV remain difficult to profile as the amount of available protein is usually very low when compared to cells or tissues. Thus, good profiling depth may be hard to achieve since some analysis workflows become unsuited to the task (e.g. ‘offline’ sample complexity reduction methods).

## 8 SUMMARY

MS‐based profiling of the sEV proteome provides a unique view of the status of the cells of interest. It may be used as a surrogate of its proteome, but this comes with several shortcomings. Isolation methods impacting the proteomic profile, no sEV‐specific internal standard, high heterogenicity, lack of distinction between disease‐specific and stress‐related markers, and biological contamination remain the main limitations of this field. Although new workflows of MS proteomic analyses are developed, these are most often prepared with cells or tissues in mind. This leaves sEV proteomics as a promising tool in the search for biomarkers and the study of cellular communication, but a multitude of challenges should be acknowledged and addressed in future studies.

Proteomic profiling of small extracellular vesicles using MS‐based methods enables researchers to obtain information about a wide array of sEV proteins and their abundance. This process is complex and thus depends on a set of factors that should be recognized before an analysis takes place. Multiple studies have been published comparing isolation methods and some provide information about the sEV source quality criteria, for example, the need to use sEV‐depleted FBS in cell culture. On the other hand, minimal or no information is provided as to the subsequent steps in this process. Such steps include but are not necessarily limited to vesicle lysis and protein solubilization, protein digestion, complexity reduction (either ‘offline’ or ‘online’), MS analysis, and its type and data analysis. These elements have been described and compared as to their performance for cells or tissue samples, but no specific information is given to the sEV samples which differ in terms of their complexity and protein content. Due to the lack of such information, results which are obtained for cells or tissues are often ‘extrapolated’ to sEV samples and this may not provide reliable results. It is especially important in terms of sEV protein content and contaminants, which differ substantially between sEV and cells or tissues.

## AUTHOR CONTRIBUTIONS

**Daniel Fochtman**: Conceptualization (equal); writing—original draft (equal); writing—review and editing (equal). **Monika Pietrowska**: Conceptualization (equal); writing—review and editing (equal). **Lukasz Marczak**: Conceptualization (equal); Writing—review & editing (equal). **Anna Wojakowska**: Conceptualization (equal); supervision (equal); writing—review and editing (equal).

## CONFLICT OF INTEREST STATEMENT

The authors declare no conflict of interest.

## References

[jev270020-bib-0001] Abcam Lyophilized Exosome Standard (Human Plasma) ab288118 . https://www.abcam.com/en‐us/products/reagents/lyophilized‐exosome‐standard‐human‐plasma‐ab288118

[jev270020-bib-0002] Abramowicz, A. , Marczak, L. , Wojakowska, A. , Zapotoczny, S. , Whiteside, T. L. , Widlak, P. , & Pietrowska, M. (2018). Harmonization of exosome isolation from culture supernatants for optimized proteomics analysis. PLOS ONE, 13, e0205496. 10.1371/journal.pone.0205496 30379855 PMC6209201

[jev270020-bib-0003] Ahmadi, S. , & Winter, D. (2018). Identification of poly(ethylene glycol) and poly(ethylene glycol)‐based detergents using peptide search engines. Analytical Chemistry, 90, 6594–6600. 10.1021/acs.analchem.8b00365 29726681

[jev270020-bib-0004] Alvarez, S. , Suazo, C. , Boltansky, A. , Ursu, M. , Carvajal, D. , Innocenti, G. , Vukusich, A. , Hurtado, M. , Villanueva, S. , Carreño, J. E. , Rogelio, A. , & Irarrazabal, C. E. (2013). Urinary exosomes as a source of kidney dysfunction biomarker in renal transplantation. Transplantation Proceedings, 45, 3719–3723. 10.1016/j.transproceed.2013.08.079 24315007

[jev270020-bib-0005] An, M. , Lohse, I. , Tan, Z. , Zhu, J. , Wu, J. , Kurapati, H. , Morgan, M. A. , Lawrence, T. S. , Cuneo, K. C. , & Lubman, D. M. (2017). Quantitative proteomic analysis of serum exosomes from patients with locally advanced pancreatic cancer undergoing chemoradiotherapy. Journal of Proteome Research, 16, 1763–1772. 10.1021/acs.jproteome.7b00024 28240915 PMC5462613

[jev270020-bib-0006] Askenase, P. W. (2022). Exosome carrier effects; resistance to digestion in phagolysosomes may assist transfers to targeted cells; II transfers of miRNAs are better analyzed via systems approach as they do not fit conventional reductionist stoichiometric concepts. International Journal of Molecular Sciences, 23, 6192. 10.3390/ijms23116192 35682875 PMC9181154

[jev270020-bib-0007] Baker, C. P. , Bruderer, R. , Abbott, J. , Arthur, J. S. C. , & Brenes, A. J. (2024). Optimizing spectronaut search parameters to improve data quality with minimal proteome coverage reductions in DIA analyses of heterogeneous samples. Journal of Proteome Research, 23, 1926–1936. 10.1021/acs.jproteome.3c00671 38691771 PMC11165578

[jev270020-bib-0008] Barberis, E. , Vanella, V V. , Falasca, M. , Caneapero, V. , Cappellano, G. , Raineri, D. , Ghirimoldi, M. , De Giorgis, V. , Puricelli, C. , Vaschetto, R. , Sainaghi, P. P. , Bruno, S. , Sica, A. , Dianzani, U. , Rolla, R. , Chiocchetti, A. , Cantaluppi, V. , Baldanzi, G. , Marengo, E. , & Manfredi, M. (2021). Circulating exosomes are strongly involved in SARS‐CoV‐2 infection. Frontiers in Molecular Biosciences, 8, 63229. 10.3389/fmolb.2021.632290 PMC7937875 33693030

[jev270020-bib-0009] Bekker‐Jensen, D B. , Martínez‐Val, A. , Steigerwald, S. , Rüther, P. , Fort, K L. , Arrey, T N. , Harder, A. , Makarov, A. , & Olsen, J V. (2020). A compact quadrupole‐orbitrap mass spectrometer with FAIMS interface improves proteome coverage in short LC gradients. Molecular & Cellular Proteomics: MCP, 19, 716–729. 10.1074/mcp.TIR119.001906 32051234 PMC7124470

[jev270020-bib-0010] Bellei, E. , Bergamini, S. , Monari, E. , Fantoni, L. I. , Cuoghi, A. , Ozben, T. , & Tomasi, A. (2011). High‐abundance proteins depletion for serum proteomic analysis: Concomitant removal of non‐targeted proteins. Amino Acids, 40, 145–156. 10.1007/s00726-010-0628-x 20495836

[jev270020-bib-0011] Beninson, L. A. , & Fleshner, M. (2014). Exosomes: An emerging factor in stress‐induced immunomodulation. Seminars in Immunology, 26(5), 394–401. 10.1016/j.smim.2013.12.001 24405946

[jev270020-bib-0012] Björhall, K. , Miliotis, T. , & Davidsson, P. (2005). Comparison of different depletion strategies for improved resolution in proteomic analysis of human serum samples. Proteomics, 5, 307–317. 10.1002/pmic.200400900 15619298

[jev270020-bib-0013] Blijdorp, C. J. , Tutakhel, O. A. Z. , Hartjes, T. A. , Van Den Bosch, T. P. P. , Van Heugten, M. H. , Rigalli, J. P. , Willemsen, R. , Musterd‐Bhaggoe, U. M. , Barros, E. R. , Carles‐Fontana, R. , Carvajal, C. A. , Arntz, O. J. , Van De Loo, F. A. J. , Jenster, G. , Clahsen‐Van Groningen, M. C. , Cuevas, C. A. , Severs, D. , Fenton, R. A. , Van Royen, M. E. , & Hoorn, E. J. (2021). Comparing approaches to normalize, quantify, and characterize urinary extracellular vesicles. Journal of the American Society of Nephrology: JASN, 32(5), 10.1681/ASN.2020081142 PMC8259679 33782168

[jev270020-bib-0014] Borberg, E. , Pashko, S. , Koren, V. , Burstein, L. , & Patolsky, F. (2021). Depletion of highly abundant protein species from biosamples by the use of a branched silicon nanopillar on‐chip platform. Analytical Chemistry, 93, 14527–14536. 10.1021/acs.analchem.1c03506 34668374 PMC8592501

[jev270020-bib-0015] Braun, F. , Rinschen, M. , Buchner, D. , Bohl, K. , Plagmann, I. , Bachurski, D. , Richard Späth, M. , Antczak, P. , Göbel, H. , Klein, C. , Lackmann, J.‐W. , Kretz, O. , Puelles, V. G. , Wahba, R. , Hallek, M. , Schermer, B. , Benzing, T. , Huber, T. B. , Beyer, A. , … Müller, R.‐U. (2020). The proteomic landscape of small urinary extracellular vesicles during kidney transplantation. Journal of Extracellular Vesicles, 10, 1–21. 10.1002/jev2.12026 PMC7710132 33304478

[jev270020-bib-0016] Brennan, K. , Martin, K. , Fitzgerald, S. P. , O'sullivan, J. , Wu, Y. , Blanco, A. , Richardson, C. , & Mc Gee, M. M. (2020). A comparison of methods for the isolation and separation of extracellular vesicles from protein and lipid particles in human serum. Scientific Reports, 10(1), 10.1038/s41598-020-57497-7 PMC6978318 31974468

[jev270020-bib-0017] Brown, K A. , Chen, B. , Guardado‐Alvarez, T M. , Lin, Z. , Hwang, L. , Ayaz‐Guner, S. , Jin, S. , & Ge, Y. (2019). A photocleavable surfactant for top‐down proteomics. Nature Methods, 16, 417–420. 10.1038/s41592-019-0391-1 30988469 PMC6532422

[jev270020-bib-0018] Bruker Daltonics . TimsTOF Pro. The new standard for high speed, high sensitivity shotgun proteomics .

[jev270020-bib-0019] Bruker Daltonics . TimsTOF Pro2. The new standard for high speed, high sensitivity 4D‐multiomics .

[jev270020-bib-0020] Brzhozovskiy, A. , Kononikhin, A. , Bugrova, A E. , Kovalev, G I. , Schmit, P.‐O. , Kruppa, G. , Nikolaev, E N. , & Borchers, C H. (2022). The parallel reaction monitoring‐parallel accumulation–serial fragmentation (prm‐PASEF) approach for multiplexed absolute quantitation of proteins in human plasma. Analytical Chemistry, 94, 2016–2022. 10.1021/acs.analchem.1c03782 35040635

[jev270020-bib-0021] Buck, K. M. , Roberts, D. S. , Aballo, T. J. , Inman, D. R. , Jin, S. , Ponik, S. , Brown, K. A. , & Ge, Y. (2022). One‐pot exosome proteomics enabled by a photocleavable surfactant. Analytical Chemistry, 94, 7164–7168. 10.1021/acs.analchem.2c01252 35543580 PMC9297302

[jev270020-bib-0022] Burkova, E E. , Grigor'eva, A E. , Bulgakov, D V. , Dmitrenok, P S. , Vlassov, V V. , Ryabchikova, E I. , Sedykh, S E. , & Nevinsky, G A. (2019). Extra purified exosomes from human placenta contain an unpredictable small number of different major proteins. International Journal of Molecular Sciences, 20, 2434. 10.3390/ijms20102434 31100946 PMC6566543

[jev270020-bib-0023] Cao, B. , Wang, P. , Gu, L. , & Liu, J. (2021). Use of four genes in exosomes as biomarkers for the identification of lung adenocarcinoma and lung squamous cell carcinoma. Oncology Letters, 21, 10.3892/ol.2021.12510 PMC7882885 33664813

[jev270020-bib-0024] Carnino, J M. , Ni, K. , & Jin, Y. (2020). Post‐translational modification regulates formation and cargo‐loading of extracellular vesicles. Frontiers in Immunology, 11, 948. 10.3389/fimmu.2020.00948 32528471 PMC7257894

[jev270020-bib-0025] Chen, T. , Xie, M.‐Y. , Sun, J.‐J. , Ye, R.‐S. , Cheng, X. , Sun, R.‐P. , Wei, Li‐M. , Li, M. , Lin, D.‐L. , Jiang, Q.‐Y. , Xi, Q.‐Y. , & Zhang, Y.‐L. (2016). Porcine milk‐derived exosomes promote proliferation of intestinal epithelial cells. Scientific Reports, 6, 33862. 10.1038/srep33862 27646050 PMC5028765

[jev270020-bib-0026] Cheruiyot, C. , Pataki, Z. , Williams, R. , Ramratnam, B. , & Li, M. (2017). SILAC based proteomic characterization of exosomes from HIV‐1 infected cells. Journal of Visualized Experiments: JoVE, 121, 54799. 10.3791/54799 PMC5409197 28287540

[jev270020-bib-0027] Chitti, S. V. , Gummadi, S. , Kang, T. , Shahi, S. , Marzan, A. L. , Nedeva, C. , Sanwlani, R. , Bramich, K. , Stewart, S. , Petrovska, M. , Sen, B. , Ozkan, A. , Akinfenwa, M. , Fonseka, P. , & Mathivanan, S. (2024). Vesiclepedia 2024: An extracellular vesicles and extracellular particles repository. Nucleic Acids Research, 52(D1), D1694–D1698. 10.1093/nar/gkad1007 37953359 PMC10767981

[jev270020-bib-0028] Cho, K.‐C. , Clark, D J. , Schnaubelt, M. , Teo, G. C. , Leprevost, F. D. V. , Bocik, W. , Boja, E. S. , Hiltke, T. , Nesvizhskii, A. I. , & Zhang, H. (2020). Deep proteomics using two dimensional data independent acquisition mass spectrometry. Analytical Chemistry, 92, 4217–4225. 10.1021/acs.analchem.9b04418 32058701 PMC7255061

[jev270020-bib-0029] Cho, S. , Yang, H. C. , & Rhee, W. J. (2020). Development and comparative analysis of human urine exosome isolation strategies. Process Biochemistry, 88, 197–203. 10.1016/j.procbio.2019.09.017

[jev270020-bib-0030] Clark, D J. , Fondrie, W E. , Yang, A. , & Mao, L. (2016). Triple SILAC quantitative proteomic analysis reveals differential abundance of cell signaling proteins between normal and lung cancer‐derived exosomes. Journal of Proteomics, 133, 161–169. 10.1016/j.jprot.2015.12.023 26739763

[jev270020-bib-0031] Cox, J. , Hein, M Y. , Luber, C A. , Paron, I. , Nagaraj, N. , & Mann, M. (2014). Accurate proteome‐wide label‐free quantification by delayed normalization and maximal peptide ratio extraction, termed MaxLFQ. Molecular & Cellular Proteomics: MCP, 13, 2513–2526. 10.1074/mcp.M113.031591 24942700 PMC4159666

[jev270020-bib-0032] Cox, J. , & Mann, M. (2008). MaxQuant enables high peptide identification rates, individualized p.p.b.‐range mass accuracies and proteome‐wide protein quantification. Nature Biotechnology, 26, 1367–1372. 10.1038/nbt.1511 19029910

[jev270020-bib-0033] cRAP ‐ The common Repository of Adventitious Proteins . https://www.thegpm.org/crap/

[jev270020-bib-0034] Creative Biolabs Exosome Standards . https://www.creative‐biolabs.com/exosome/category‐exosome‐standards‐53.htm

[jev270020-bib-0035] Dau, T. , Bartolomucci, G. , & Rappsilber, J. (2020). Proteomics using protease alternatives to trypsin benefits from sequential digestion with trypsin. Analytical Chemistry, 92, 9523–9527. 10.1021/acs.analchem.0c00478 32628831 PMC7377536

[jev270020-bib-0036] Demichev, V. , Messner, C B. , Vernardis, S I. , Lilley, K S. , & Ralser, M. (2020). DIA‐NN: Neural networks and interference correction enable deep proteome coverage in high throughput. Nature Methods, 17, 41–44. 10.1038/s41592-019-0638-x 31768060 PMC6949130

[jev270020-bib-0037] Ding, R. , Liu, Z. , Wang, J. , Xia, T. , & Li, L. (2024). DIA‐based quantitative proteomics analysis of plasma exosomes in rat model of allergic rhinitis. Analytical Biochemistry, 688, 115463. 10.1016/j.ab.2024.115463 38244750

[jev270020-bib-0038] Ding, X.‐Q. , Wang, Z.‐Y. , Xia, D. , Wang, R.‐X. , Pan, X.‐R. , & Tong, J.‐H. (2020). Proteomic profiling of serum exosomes from patients with metastatic gastric cancer. Frontiers in Oncology, 10, 1113. 10.3389/fonc.2020.01113 32754443 PMC7367030

[jev270020-bib-0039] Dubois, E. , Galindo, A. N. , Dayon, L. , & Cominetti, O. (2022). Assessing normalization methods in mass spectrometry‐based proteome profiling of clinical samples. Biosystems, 215–216, 104661. 10.1016/j.biosystems.2022.104661 35247480

[jev270020-bib-0040] Duncan, M. W. , Nedelkov, D. , Walsh, R. , & Hattan, S. J. (2016). Applications of MALDI mass spectrometry in clinical chemistry. Clinical Chemistry, 62, 134–143. 10.1373/clinchem.2015.239491 26585930

[jev270020-bib-0041] Eldrid, C. , Ujma, J. , Kalfas, S. , Tomczyk, N. , Giles, K. , Morris, M. , & Thalassinos, K. (2019). Gas phase stability of protein ions in a cyclic ion mobility spectrometry traveling wave device. Analytical Chemistry, 91, 7554–7561. 10.1021/acs.analchem.8b05641 31117399 PMC7006968

[jev270020-bib-0042] Eliuk, S. , & Makarov, A. (2015). Evolution of orbitrap mass spectrometry instrumentation. Annual Review of Analytical Chemistry, 8, 61–80. 10.1146/annurev-anchem-071114-040325 26161972

[jev270020-bib-0043] Exosome Gene Ontology Annotation Initiative | European Bioinformatics Institute . https://www.ebi.ac.uk/GOA/EXOSOME

[jev270020-bib-0044] Frankenfield, A M. , Ni, J. , Ahmed, M. , & Hao, L. (2022). Protein contaminants matter: Building universal protein contaminant libraries for DDA and DIA proteomics. Journal of Proteome Research, 21, 2104–2113. 10.1021/acs.jproteome.2c00145 35793413 PMC10040255

[jev270020-bib-0045] Fricke, F. , Michalak, M. , Warnken, U. , Hausser, I. , Schnölzer, M. , Kopitz, J. , & Gebert, J. (2019). SILAC‐based quantification of TGFBR2‐regulated protein expression in extracellular vesicles of microsatellite unstable colorectal cancers. International Journal of Molecular Sciences, 20, 4162. 10.3390/ijms20174162 31454892 PMC6747473

[jev270020-bib-0046] Fröhlich, K. , Brombacher, E. , Fahrner, M. , Vogele, D. , Kook, L. , Pinter, N. , Bronsert, P. , Timme‐Bronsert, S. , Schmidt, A. , Bärenfaller, K. , Kreutz, C. , & Schilling, O. (2022). Benchmarking of analysis strategies for data‐independent acquisition proteomics using a large‐scale dataset comprising inter‐patient heterogeneity. Nature Communications, 13, 10.1038/s41467-022-30094-0 PMC9098472 35551187

[jev270020-bib-0047] Fujita, K. , Kume, H. , Matsuzaki, K. , Kawashima, A. , Ujike, T. , Nagahara, A. , Uemura, M. , Miyagawa, Y. , Tomonaga, T. , & Nonomura, N. (2017). Proteomic analysis of urinary extracellular vesicles from high Gleason score prostate cancer. Scientific Reports, 7, 42961. 10.1038/srep42961 28211531 PMC5314323

[jev270020-bib-0048] Garcia‐Martin, R. , Brandao, B. B. , Thomou, T. , Altindis, E. , & Kahn, C. R (2022). Tissue differences in the exosomal/small extracellular vesicle proteome and their potential as indicators of altered tissue metabolism. Cell Reports, 38, 110277. 10.1016/j.celrep.2021.110277 35045290 PMC8867597

[jev270020-bib-0049] Gawin, M. , Wojakowska, A. , Pietrowska, M. , Marczak, Ł. , Chekan, M. , Jelonek, K. , Lange, D. , Jaksik, R. , Gruca, A. , & Widłak, P. (2018). Proteome profiles of different types of thyroid cancers. Molecular and Cellular Endocrinology, 472, 68–79. 10.1016/j.mce.2017.11.020 29183805

[jev270020-bib-0050] Geddes‐Mcalister, J. , & Gadjeva, M. (2019). Mass spectrometry‐based quantitative proteomics of murine‐derived polymorphonuclear neutrophils. Current Protocols in Immunology, 126(1), e87. 10.1002/cpim.87 31483107 PMC6730563

[jev270020-bib-0051] Gessulat, S. , Schmidt, T. , Zolg, D. P. , Samaras, P. , Schnatbaum, K. , Zerweck, J. , Knaute, T. , Rechenberger, J. , Delanghe, B. , Huhmer, A. , Reimer, U. , Ehrlich, H.‐C. , Aiche, S. , Kuster, B. , & Wilhelm, M. (2019). Prosit: Proteome‐wide prediction of peptide tandem mass spectra by deep learning. Nature Methods, 16, 509–518. 10.1038/s41592-019-0426-7 31133760

[jev270020-bib-0052] Gillette, M. A. , & Carr, S. A. (2013). Quantitative analysis of peptides and proteins in biomedicine by targeted mass spectrometry. Nature Methods, 10, 28–34. 10.1038/nmeth.2309 23269374 PMC3943160

[jev270020-bib-0053] Giri, P K. , Kruh, N A. , Dobos, K M. , & Schorey, J S. (2010). Proteomic analysis identifies highly antigenic proteins in exosomes from M. tuberculosis‐infected and culture filtrate protein‐treated macrophages. Proteomics, 10, 3190–3202. 10.1002/pmic.200900840 20662102 PMC3664454

[jev270020-bib-0054] Glatter, T. , Ahrné, E. , & Schmidt, A. (2015). Comparison of different sample preparation protocols reveals lysis buffer‐specific extraction biases in gram‐negative bacteria and human cells. Journal of Proteome Research, 14, 4472–4485. 10.1021/acs.jproteome.5b00654 26412744

[jev270020-bib-0055] Gołębiewska, J E. , Wardowska, A. , Pietrowska, M. , Wojakowska, A. , & Dębska‐Ślizień, A. (2021). Small extracellular vesicles in transplant rejection. Cells, 10, 2989. 10.3390/cells10112989 34831212 PMC8616261

[jev270020-bib-0056] Gonzales, P A. , Pisitkun, T. , Hoffert, J D. , Tchapyjnikov, D. , Star, R A. , Kleta, R. , Wang, N. S. , & Knepper, M A. (2009). Large‐scale proteomics and phosphoproteomics of urinary exosomes. Journal of the American Society of Nephrology: JASN, 20(2), 363–379. 10.1681/ASN.2008040406 19056867 PMC2637050

[jev270020-bib-0057] Han, Z. , Peng, C. , Yi, J. , Wang, Y. , Liu, Q. , Yang, Y. , Long, S. , Qiao, L. , & Shen, Y. (2021). Matrix‐assisted laser desorption ionization mass spectrometry profiling of plasma exosomes evaluates osteosarcoma metastasis. iScience, 24(8), 102906. 10.1016/j.isci.2021.102906 34401680 PMC8355924

[jev270020-bib-0058] Harmati, M. , Gyukity‐Sebestyen, E. , Dobra, G. , Janovak, L. , Dekany, I. , Saydam, O. , Hunyadi‐Gulyas, E. , Nagy, I. , Farkas, A. , Pankotai, T. , Ujfaludi, Z. , Horvath, P. , Piccinini, F. , Kovacs, M. , Biro, T. , & Buzas, K. (2019). Small extracellular vesicles convey the stress‐induced adaptive responses of melanoma cells. Scientific Reports, 9(1), 15329. 10.1038/s41598-019-51778-6 31653931 PMC6814750

[jev270020-bib-0059] Heidarzadeh, M. , Zarebkohan, A. , Rahbarghazi, R. , & Sokullu, E. (2023). Protein corona and exosomes: New challenges and prospects. *Cell Communication and Signaling*, 21. 10.1186/s12964-023-01089-1 PMC10041507 36973780

[jev270020-bib-0060] Heil, L R. , Damoc, E. , Arrey, T N. , Pashkova, A. , Denisov, E. , Petzoldt, J. , Peterson, A C. , Hsu, C. , Searle, B C. , Shulman, N. , Riffle, M. , Connolly, B. , Maclean, B X. , Remes, P M. , Senko, M W. , Stewart, H. I. , Hock, C. , Makarov, A A. , Hermanson, D. , … Maccoss, M J. (2023). Evaluating the performance of the astral mass analyzer for quantitative proteomics using data‐independent acquisition. Journal of Proteome Research, 22, 3290–3300. 10.1021/acs.jproteome.3c00357 37683181 PMC10563156

[jev270020-bib-0061] Hoshino, A. , Kim, H. S. , Bojmar, L. , Gyan, K. E. , Cioffi, M. , Hernandez, J. , Zambirinis, C P. , Rodrigues, G. , Molina, H. , Heissel, S. , Mark, M. T. , Steiner, L. , Benito‐Martin, A. , Lucotti, S. , Di Giannatale, A. , Offer, K. , Nakajima, M. , Williams, C. , Nogués, L. , … Lyden, D. (2020). Extracellular vesicle and particle biomarkers define multiple human cancers. Cell, 182, 1044–1061. e18. 10.1016/j.cell.2020.07.009 32795414 PMC7522766

[jev270020-bib-0062] Huang, Y. , Liu, Y. , Huang, Q. , Sun, S. , Ji, Z. , Huang, L. , Li, Z. , Huang, X. , Deng, W. , & Li, T. (2022). TMT‐based quantitative proteomics analysis of synovial fluid‐derived exosomes in inflammatory arthritis. Frontiers in Immunology, 13, 800902. 10.3389/fimmu.2022.800902 35359923 PMC8961740

[jev270020-bib-0063] Hughes, C S. , Moggridge, S. , Müller, T. , Sorensen, P H. , Morin, G B. , & Krijgsveld, J. (2019). Single‐pot, solid‐phase‐enhanced sample preparation for proteomics experiments. Nature Protocols, 14, 68–85. 10.1038/s41596-018-0082-x 30464214

[jev270020-bib-0064] Hurwitz, S N. , Rider, M A. , Bundy, J L. , Liu, X. , Singh, R K. , & Meckes, D G. (2016). Proteomic profiling of NCI‐60 extracellular vesicles uncovers common protein cargo and cancer type‐specific biomarkers. Oncotarget, 7, 86999–87015. 10.18632/oncotarget.13569 27894104 PMC5341331

[jev270020-bib-0065] Hüttmann, N. , Li, Y. , Poolsup, S. , Zaripov, E. , D'mello, R. , Susevski, V. , Minic, Z. , & Berezovski, M V. (2024). Surface proteome of extracellular vesicles and correlation analysis reveal breast cancer biomarkers. Cancers, 16, 520. 10.3390/cancers16030520 38339272 PMC10854524

[jev270020-bib-0066] Ignatoski, K. M. W. , & Verderame, M. F. (1996). Lysis buffer composition dramatically affects extraction of phosphotyrosine‐containing proteins. BioTechniques, 20, 794–796. 10.2144/96205bm13 8723920

[jev270020-bib-0067] Ilavenil, S. , Al‐Dhabi, N. A. , Srigopalram, S. , Kim, Y. O. , Agastian, P. , Baaru, R. , Choi, K. C. , Arasu, M. V. , Park, C. G. , & Park, K. H. (2016). Removal of SDS from biological protein digests for proteomic analysis by mass spectrometry, *Proteome Science*, *14*. 10.1186/s12953-016-0098-5 PMC5012027 27601941

[jev270020-bib-0068] Immunostep Biotech. Lyophilized Exosome Standards . Immunostep Biotech . https://immunostep.com/exosomes/lyophilized‐exosome‐standards/

[jev270020-bib-0069] Jaros, J. A. J. , Guest, P. C. , Bahn, S. , & Martins‐de‐Souza, D. (2013). Affinity depletion of plasma and serum for mass spectrometry‐based proteome analysis, In M. Zhou & T. Veenstra (Eds.), Proteomics for biomarker discovery, Humana Press, pp. 1–11. 10.1007/978-1-62703-360-2_1 23625390

[jev270020-bib-0070] Jiang, R. , Rong, C. , Ke, R. , Meng, S. , Yan, X. , Ke, H. , & Wu, S. (2019). Differential proteomic analysis of serum exosomes reveals alterations in progression of Parkinson disease. Medicine, 98(41), e17478. 10.1097/MD.0000000000017478 31593110 PMC6799836

[jev270020-bib-0071] Jimenez, D. E. , Tahir, M. , Faheem, M. , Alves, W. B. D. S. , Correa, B. L. , Andrade, G. R. , Larsen, M. R. , Oliveira, G. P. Jr , & Pereira, R. W. (2023). Comparison of four purification methods on serum extracellular vesicle recovery, size distribution, and proteomics. Proteomes, 11(3), 23. 10.3390/proteomes11030023 37606419 PMC10443378

[jev270020-bib-0072] Jung, H. H. , Kim, J.‐Y. , Lim, J. E. , & Im, Y.‐H. (2020). Cytokine profiling in serum‐derived exosomes isolated by different methods. Scientific Reports, 10(1), 14069. 10.1038/s41598-020-70584-z 32826923 PMC7442638

[jev270020-bib-0073] Kalluri, R. , & LeBleu, V. S. (2020). The biology, function, and biomedical applications of exosomes. *Science (New York, N.Y.)*, *367* 10.1126/science.aau6977 PMC7717626 32029601

[jev270020-bib-0074] Kangas, P. , Nyman, T A. , Metsähonkala, L. , Burns, C. , Tempest, R. , Williams, T. , Karttunen, J. , & Jokinen, T S. (2023). Towards optimised extracellular vesicle proteomics from cerebrospinal fluid. Scientific Reports, 13, 9564. 10.1038/s41598-023-36706-z 37308520 PMC10261101

[jev270020-bib-0075] Karp, N. A. , Huber, W. , Sadowski, P. G. , Charles, P. D. , Hester, S. V. , & Lilley, K. S. (2010). Addressing accuracy and precision issues in iTRAQ quantitation. Molecular & Cellular Proteomics, 9(9), 1885–1897. 10.1074/mcp.M900628-MCP200 20382981 PMC2938101

[jev270020-bib-0076] Keerthikumar, S. , Chisanga, D. , Ariyaratne, D. , Al Saffar, H. , Anand, S. , Zhao, K. , Samuel, M. , Pathan, M. , Jois, M. , Chilamkurti, N. , Gangoda, L. , & Mathivanan, S. (2016). ExoCarta: A web‐based compendium of exosomal cargo. Journal of Molecular Biology, 428, 688–692. 10.1016/J.JMB.2015.09.019 26434508 PMC4783248

[jev270020-bib-0077] Keller, B O. , Sui, J. , Young, A B. , & Whittal, R M. (2008). Interferences and contaminants encountered in modern mass spectrometry. Analytica Chimica Acta, 627, 71–81. 10.1016/j.aca.2008.04.043 18790129

[jev270020-bib-0078] Kong, A. T. , Leprevost, F. V. , Avtonomov, D. M. , Mellacheruvu, D. , & Nesvizhskii, A. I. (2017). MSFragger: Ultrafast and comprehensive peptide identification in mass spectrometry‐based proteomics. Nature Methods, 14, 513–520. 10.1038/nmeth.4256 28394336 PMC5409104

[jev270020-bib-0079] Koritzinsky, E H. , Street, J M. , Star, R A. , & Yuen, P. S. T. (2017). Quantification of exosomes. Journal of Cellular Physiology, 232, 1587–1590. 10.1002/jcp.25387 27018079 PMC5039048

[jev270020-bib-0080] Krüger, M. , Moser, M. , Ussar, S. , Thievessen, I. , Luber, C A. , Forner, F. , Schmidt, S. , Zanivan, S. , Fässler, R. , & Mann, M. (2008). SILAC mouse for quantitative proteomics uncovers kindlin‐3 as an essential factor for red blood cell function. Cell, 134, 353–364. 10.1016/j.cell.2008.05.033 18662549

[jev270020-bib-0081] Kugeratski, F G. , Hodge, K. , Lilla, S. , Mcandrews, K M. , Zhou, X. , Hwang, R F. , Zanivan, S. , & Kalluri, R. (2021). Quantitative proteomics identifies the core proteome of exosomes with syntenin‐1 as the highest abundant protein and a putative universal biomarker. Nature Cell Biology, 23, 631–641. 10.1038/s41556-021-00693-y 34108659 PMC9290189

[jev270020-bib-0082] Kw, P. , & Kierulf, B. (2015). Direct Isolation of exosomes from cell culture: Simplifying methods for exosome enrichment and analysis. Translational Biomedicine, 17(1), 6. 10.21767/2172-0479.100018

[jev270020-bib-0083] Lattmann, E. , Räss, L. , Tognetti, M. , Gómez, J M. M , Lapaire, V. , Bruderer, R. , Reiter, L. , Feng, Y. , Steinmetz, L M. , & Levesque, M P. (2024). Size‐exclusion chromatography combined with DIA‐MS enables deep proteome profiling of extracellular vesicles from melanoma plasma and serum. Cellular and Molecular Life Sciences, 81(1), 90. 10.1007/s00018-024-05137-y 38353833 PMC10867102

[jev270020-bib-0084] Le Gall, L. , Ouandaogo, Z. G. , Anakor, E. , Connolly, O. , Butler Browne, G. , Laine, J. , Duddy, W. , & Duguez, S. (2020). Optimized method for extraction of exosomes from human primary muscle cells. Skeletal Muscle, 10(1), 20. 10.1186/s13395-020-00238-1 32641118 PMC7341622

[jev270020-bib-0085] Lehrich, B M. , Liang, Y. , & Fiandaca, M S. (2021). Foetal bovine serum influence on in vitro extracellular vesicle analyses. Journal of Extracellular Vesicles, 10(3), e12061. 10.1002/jev2.12061 33532042 PMC7830136

[jev270020-bib-0086] Li, J. , Smith, L S. , & Zhu, H.‐J. (2021). Data‐independent acquisition (DIA): An emerging proteomics technology for analysis of drug‐metabolizing enzymes and transporters. Drug Discovery Today: Technologies, 39, 49–56. 10.1016/j.ddtec.2021.06.006 34906325 PMC8674493

[jev270020-bib-0087] Li, R. , & Shen, Y. (2013). An old method facing a new challenge: Re‐visiting housekeeping proteins as internal reference control for neuroscience research. Life Sciences, 92, 747–751. 10.1016/j.lfs.2013.02.014 23454168 PMC3614345

[jev270020-bib-0088] Li, S. , Li, X. , Yang, S. , Pi, H. , Li, Z. , Yao, P. , Zhang, Q. , Wang, Q. , Shen, P. , Li, X. , & Ji, J. (2021). Proteomic landscape of exosomes reveals the functional contributions of CD151 in triple‐negative breast cancer. Molecular & Cellular Proteomics, 20, 100121. 10.1016/j.mcpro.2021.100121 34265469 PMC8379346

[jev270020-bib-0089] Li, S. , Li, X. , Yang, S. , Pi, H. , Li, Z. , Yao, P. , Zhang, Q. , Wang, Q. , Shen, P. , Li, X. , & Ji, J. (2021). Proteomic landscape of exosomes reveals the functional contributions of CD151 in triple‐negative breast cancer. Molecular & Cellular Proteomics: MCP, 20, 100121. 10.1016/j.mcpro.2021.100121 34265469 PMC8379346

[jev270020-bib-0090] Li, S. , Yi, M. , Dong, B. , Tan, X. , Luo, S. , & Wu, K. (2021). The role of exosomes in liquid biopsy for cancer diagnosis and prognosis prediction. International Journal of Cancer, 148(11), 2640–2651. 10.1002/ijc.33386 33180334 PMC8049049

[jev270020-bib-0091] Lou, R. , Cao, Y. , Li, S. , Lang, X. , Li, Y. , Zhang, Y. , & Shui, W. (2023). Benchmarking commonly used software suites and analysis workflows for DIA proteomics and phosphoproteomics. Nature Communications, 14(1), 94. 10.1038/s41467-022-35740-1 PMC9822986 36609502

[jev270020-bib-0092] Ludwig, K R. , Schroll, M M. , & Hummon, A B. (2018). Comparison of in‐solution, FASP, and S‐trap based digestion methods for bottom‐up proteomic studies. Journal of Proteome Research, 17, 2480–2490. 10.1021/acs.jproteome.8b00235 29754492 PMC9319029

[jev270020-bib-0093] Ludwig, N. , Yerneni, S S. , Azambuja, J H. , Pietrowska, M. , Widłak, P. , Hinck, C S. , Głuszko, A. , Szczepański, M J. , Kärmer, T. , Kallinger, I. , Schulz, D. , Bauer, R J. , Spanier, G. , Spoerl, S. , Meier, J K. , Ettl, T. , Razzo, B M. , Reichert, T E. , Hinck, A P. , & Whiteside, T L. (2022). TGFβ ^+^ small extracellular vesicles from head and neck squamous cell carcinoma cells reprogram macrophages towards a pro‐angiogenic phenotype. Journal of Extracellular Vesicles, 11, e12294. 10.1002/jev2.12294 36537293 PMC9764108

[jev270020-bib-0094] Madern, M. , Reiter, W. , Stanek, F. , Hartl, N. , Mechtler, K. , & Hartl, M. (2024). A causal model of ion interference enables assessment and correction of ratio compression in multiplex proteomics. Molecular & Cellular Proteomics, 23, 100694. 10.1016/j.mcpro.2023.100694 38097181 PMC10828822

[jev270020-bib-0095] Makler, A. , & Asghar, W. (2020). Exosomal biomarkers for cancer diagnosis and patient monitoring. Expert Review of Molecular Diagnostics, 20(4), 387–400. 10.1080/14737159.2020.1731308 32067543 PMC7071954

[jev270020-bib-0096] Malinowska, A. , Kistowski, M. , Bakun, M. , Rubel, T. , Tkaczyk, M. , Mierzejewska, J. , & Dadlez, M. (2012). Diffprot—Software for non‐parametric statistical analysis of differential proteomics data. Journal of Proteomics, 75, 4062–4073. 10.1016/j.jprot.2012.05.030 22641154

[jev270020-bib-0097] Mathew, B. , Mansuri, M. S , Williams, K R. , & Nairn, A C. (2021). Exosomes as emerging biomarker tools in neurodegenerative and neuropsychiatric disorders—A proteomics perspective. Brain Sciences, 11, 258, 10.3390/brainsci11020258 33669482 PMC7922222

[jev270020-bib-0098] May, J C. , Leaptrot, K L. , Rose, B S. , Moser, K L. W , Deng, L. , Maxon, L. , Debord, D. , & Mclean, J A. (2021). Resolving power and collision cross section measurement accuracy of a prototype high‐resolution ion mobility platform incorporating structures for lossless ion manipulation. Journal of the American Society for Mass Spectrometry, 32, 1126–1137. 10.1021/jasms.1c00056 33734709 PMC9296130

[jev270020-bib-0099] McDaniel, E. W. , Martin, D. W. , & Barnes, W. S. (2004). Drift tube‐mass spectrometer for studies of low‐energy ion‐molecule reactions. Review of Scientific Instruments, 33, 2–7. 10.1063/1.1717656

[jev270020-bib-0100] Mears, R. , Craven, R A. , Hanrahan, S. , Totty, N. , Upton, C. , Young, S L. , Patel, P. , Selby, P J. , & Banks, R E. (2004). Proteomic analysis of melanoma‐derived exosomes by two‐dimensional polyacrylamide gel electrophoresis and mass spectrometry. Proteomics, 4, 4019–4031. 10.1002/pmic.200400876 15478216

[jev270020-bib-0101] Meier, F. , Brunner, A. D. , Koch, S. , Koch, H. , Lubeck, M. , Krause, M. , Goedecke, N. , Decker, J. , Kosinski, T. , Park, M. A. , Bache, N. , Hoerning, O. , Cox, J. , Räther, O. , & Mann, M. (2018). Online parallel accumulation‐serial fragmentation (PASEF) with a novel trapped ion mobility mass spectrometer. Molecular & Cellular Proteomics: MCP, 17(12), 2534–2545. 10.1074/mcp.TIR118.000900 30385480 PMC6283298

[jev270020-bib-0102] Meng, S. , Wang, T. , Zhao, Q. , Hu, Q. , Chen, Y. , Li, H. , Liu, C. , Liu, D. , & Hong, X. (2023). Proteomics analysis of plasma‐derived exosomes unveils the aberrant complement and coagulation cascades in dermatomyositis/polymyositis. Journal of Proteome Research, 22, 123–137. 10.1021/acs.jproteome.2c00532 36507906 PMC9830643

[jev270020-bib-0103] Montero‐Calle, A. , Garranzo‐Asensio, M. , Rejas‐González, R. , Feliu, J. , Mendiola, M. , Peláez‐García, A. , & Barderas, R. (2023). Benefits of FAIMS to improve the proteome coverage of deteriorated and/or cross‐linked TMT 10‐Plex FFPE tissue and plasma‐derived exosomes samples. Proteomes, 11, 35. 10.3390/proteomes11040035 37987315 PMC10661291

[jev270020-bib-0104] Moreira‐Costa, L. , Barros, A S. , Lourenço, A P. , Leite‐Moreira, A F. , Nogueira‐Ferreira, R. , Thongboonkerd, V. , & Vitorino, R. (2021). Exosome‐derived mediators as potential biomarkers for cardiovascular diseases: A network approach. Proteomes, 9, 8. 10.3390/proteomes9010008 33535467 PMC7930981

[jev270020-bib-0105] Moreno‐Gonzalo, O. , Fernandez‐Delgado, I. , & Sanchez‐Madrid, F. (2018). Post‐translational add‐ons mark the path in exosomal protein sorting. Cellular and Molecular Life Sciences, 75, 1–19. 10.1007/s00018-017-2690-y 29080091 PMC11105655

[jev270020-bib-0106] Mrozinski, P. *et al.* (2008). Human serum and plasma protein depletion—novel high‐capacity affinity column for the removal of the “Top 14” abundant proteins.

[jev270020-bib-0107] Mutschelknaus, L. , Azimzadeh, O. , Heider, T. , Winkler, K. , Vetter, M. , Kell, R. , Tapio, S. , Merl‐Pham, J. , Huber, S M. , Edalat, L. , Radulović, V. , Anastasov, N. , Atkinson, M J. , & Moertl, S. (2017). Radiation alters the cargo of exosomes released from squamous head and neck cancer cells to promote migration of recipient cells. Scientific Reports, 7(1), 12423. 10.1038/s41598-017-12403-6 28963552 PMC5622080

[jev270020-bib-0108] Neset, L. , Takayidza, G. , Berven, F S. , & Hernandez‐Valladares, M. (2022). Comparing efficiency of lysis buffer solutions and sample preparation methods for liquid chromatography–mass spectrometry analysis of human cells and plasma. *Molecules*. 10.3390/molecules27113390 PMC9181984 35684327

[jev270020-bib-0109] Ngoka, L. C. M. (2008). Sample prep for proteomics of breast cancer: Proteomics and gene ontology reveal dramatic differences in protein solubilization preferences of radioimmunoprecipitation assay and urea lysis buffers. Proteome Science, 6, 30, 10.1186/1477-5956-6-30 18950484 PMC2600628

[jev270020-bib-0110] Ni, H. , Pan, W. , Jin, Q. , Xie, Y. , Zhang, N. , Chen, K. , Lin, T. , Lin, C. , Xie, Y. , Wu, J. , Ni, P. , & Wu, L. (2021). Label‐free proteomic analysis of serum exosomes from paroxysmal atrial fibrillation patients. Clinical Proteomics, 18, 10.1186/s12014-020-09304-8 PMC7789314 33407078

[jev270020-bib-0111] Nigjeh, E N. , Chen, R. , Brand, R E. , Petersen, G M. , Chari, S T. , Von Haller, P D. , Eng, J K. , Feng, Z. , Yan, Q. , Brentnall, T. A. , & Pan, S. (2017). Quantitative proteomics based on optimized data‐independent‐acquisition in plasma analysis. Journal of Proteome Research, 16, 665–676. 10.1021/acs.jproteome.6b00727 27995795 PMC5889294

[jev270020-bib-0112] Ogawa, Y. , Akimoto, Y. , Ikemoto, M. , Goto, Y. , Ishikawa, A. , Ohta, S. , Takase, Y. , Kawakami, H. , Tsujimoto, M. , & Yanoshita, R. (2021). Stability of human salivary extracellular vesicles containing dipeptidyl peptidase IV under simulated gastrointestinal tract conditions. Biochemistry and Biophysics Reports, 27, 101034. 10.1016/j.bbrep.2021.101034 34141904 PMC8185177

[jev270020-bib-0113] Ong, S.‐E. , Blagoev, B. , Kratchmarova, I. , Kristensen, D. B. , Steen, H. , Pandey, A. , & Mann, M. (2002). Stable isotope labeling by amino acids in cell culture, SILAC, as a simple and accurate approach to expression proteomics. Molecular & Cellular Proteomics, 1, 376–386. 10.1074/mcp.M200025-MCP200 12118079

[jev270020-bib-0114] Palomba, A. , Abbondio, M. , Fiorito, G. , Uzzau, S. , Pagnozzi, D. , & Tanca, A. (2021). Comparative evaluation of MaxQuant and proteome discoverer MS1‐based protein quantification tools. Journal of Proteome Research, 20, 3497–3507. 10.1021/acs.jproteome.1c00143 34038140 PMC8280745

[jev270020-bib-0115] Payton, C. , Pang, L Y. , Gray, M. , & Argyle, D J. (2021). Exosomes derived from radioresistant breast cancer cells promote therapeutic resistance in naïve recipient cells. Journal of Personalized Medicine, 11, 1310, 10.3390/jpm11121310 34945782 PMC8704086

[jev270020-bib-0116] Pedersen, S. , Jensen, K. P. , Honoré, B. , Kristensen, S. R. , Pedersen, C. H. , Szejniuk, W. M. , Maltesen, R. G. , & Falkmer, U. (2022). Circulating microvesicles and exosomes in small cell lung cancer by quantitative proteomics. Clinical Proteomics, 19, 10.1186/s12014-021-09339-5 PMC8903681 34996345

[jev270020-bib-0117] Picotti, P. , & Aebersold, R. (2012). Selected reaction monitoring–based proteomics: Workflows, potential, pitfalls and future directions. Nature Methods, 9, 555–566. 10.1038/nmeth.2015 22669653

[jev270020-bib-0118] Pietrowska, M. , Funk, S. , Gawin, M. , Marczak, Ł. , Abramowicz, A. , Widłak, P. , & Whiteside, T. (2017). Isolation of exosomes for the purpose of protein cargo analysis with the use of mass spectrometry, in Functional genomics, vol. 1654, Springer New York, pp. 291–307. 10.1007/978-1-4939-7231-9_22 28986800

[jev270020-bib-0119] Pino, L K. , Baeza, J. , Lauman, R. , Schilling, B. , & Garcia, B A. (2021). Improved SILAC quantification with data independent acquisition to investigate bortezomib‐induced protein degradation. Journal of Proteome Research, 20, 1918–1927. 10.1021/acs.jproteome.0c00938 33764077 PMC8256668

[jev270020-bib-0120] Pirog, A. , Faktor, J. , Urban‐Wojciuk, Z. , Kote, S. , Chruściel, E. , Arcimowicz, Ł. , Marek‐Trzonkowska, N. , Vojtesek, B. , Hupp, T R. , Al Shboul, S. , Brennan, P M. , Smoleński, R. T. , Goodlett, D R. , & Dapic, I. (2021). Comparison of different digestion methods for proteomic analysis of isolated cells and FFPE tissue samples. Talanta, 233, 122568. 10.1016/j.talanta.2021.122568 34215064

[jev270020-bib-0121] Plubell, D. L. , Remes, P. M. , Wu, C. C. , Jacob, C. C. , Merrihew, G. E. , Hsu, C. , Shulman, N. , MacLean, B. X. , Heil, L. , Poston, K. , Montine, T. , & MacCoss, M. J. (2024). Development of highly multiplex targeted proteomics assays in biofluids using the Stellar mass spectrometer. *bioRxiv*. 10.1101/2024.06.04.597431 PMC12914430 41512968

[jev270020-bib-0122] Pop, C. , Mogosan, C. , & Loghin, F. (2014). Evaluation of rapigest efficacy for the digestion of proteins from cell cultures and heart tissue. Medicine and Pharmacy Reports, 87, 258–262. 10.15386/cjmed-367 PMC4620675 26528033

[jev270020-bib-0123] Proteome Discoverer Software . https://www.thermofisher.com/uk/en/home/industrial/mass‐spectrometry/liquid‐chromatography‐mass‐spectrometry‐lc‐ms/lc‐ms‐software/multi‐omics‐data‐analysis/proteome‐discoverer‐software.html

[jev270020-bib-0124] Quirino, J. P. (2018). Sodium dodecyl sulfate removal during electrospray ionization using cyclodextrins as simple sample solution additive for improved mass spectrometric detection of peptides. Analytica Chimica Acta, 1005, 54–60. 10.1016/j.aca.2017.12.012 29389319

[jev270020-bib-0125] Rajavel, A. , Viswanathan, V. , & Murugaiyan, J. , Mariappanadar, V. (2023). Identification of extracellular vesicles derived from plasma using MALDI‐TOF MS: Influence of operating conditions. Journal of Applied Biotechnology Reports, 10(3), 1098–1108. 10.30491/jabr.2023.380590.1595

[jev270020-bib-0126] Rardin, M. J. (2018). Rapid assessment of contaminants and interferences in mass spectrometry data using skyline. Journal of The American Society for Mass Spectrometry, 29, 1327–1330. 10.1007/s13361-018-1940-z 29667163

[jev270020-bib-0127] Reinert, K. , & Kohlbacher, O. (2010). OpenMS and TOPP: Open source software for LC‐MS data analysis, in Proteome Bioinformatics, vol. 604, Humana Press, pp. 201–211. 10.1007/978-1-60761-444-9_14 20013373

[jev270020-bib-0128] Remes, P M. , Jacob, C C. , Heil, L R. , Shulman, N. , Maclean, B X. , & Maccoss, M J. (2024). Hybrid quadrupole mass filter—Radial ejection linear ion trap and intelligent data acquisition enable highly multiplex targeted proteomics. 10.1101/2024.05.31.596848 PMC11956834 39475161

[jev270020-bib-0129] Roca, L S. , Gargano, A. F. G. , & Schoenmakers, P J. (2021). Development of comprehensive two‐dimensional low‐flow liquid‐chromatography setup coupled to high‐resolution mass spectrometry for shotgun proteomics. Analytica Chimica Acta, 1156, 338349, 10.1016/j.aca.2021.338349 33781465

[jev270020-bib-0130] Romancino, D P. , Buffa, V. , Caruso, S. , Ferrara, I. , Raccosta, S. , Notaro, A. , Campos, Y. , Noto, R. , Martorana, V. , Cupane, A. , Giallongo, A. , D'azzo, A. , Manno, M. , & Bongiovanni, A. (2018). Palmitoylation is a post‐translational modification of Alix regulating the membrane organization of exosome‐like small extracellular vesicles. Biochimica et Biophysica Acta, 1862, 2879–2887. 10.1016/j.bbagen.2018.09.004 30251702

[jev270020-bib-0131] Rosa‐Fernandes, L. , Rocha, V. B. , Carregari, V. C. , Urbani, A. , & Palmisano, G. (2017). A perspective on extracellular vesicles proteomics. Frontiers in Chemistry, 5, 102. 10.3389/fchem.2017.00102 29209607 PMC5702361

[jev270020-bib-0132] Röst, H. L. , Rosenberger, G. , Navarro, P. , Gillet, L. , Miladinović, S. M. , Schubert, O. T. , Wolski, W. , Collins, B. C. , Malmström, J. , Malmström, L. , & Aebersold, R. (2014). OpenSWATH enables automated, targeted analysis of data‐independent acquisition MS data. Nature Biotechnology, 32, 219–223. 10.1038/nbt.2841 24727770

[jev270020-bib-0133] Royo, F. , Cossío, U. , Ruiz De Angulo, A. , Llop, J. , & Falcon‐Perez, J M. (2019). Modification of the glycosylation of extracellular vesicles alters their biodistribution in mice. Nanoscale, 11, 1531–1537. 10.1039/C8NR03900C 30623961

[jev270020-bib-0134] Saravanan, P. B. , Kalivarathan, J. , Khan, F. , Shah, R. , Levy, M F. , & Kanak, M A. (2023). Exosomes in transplantation: Role in allograft rejection, diagnostic biomarker, and therapeutic potential. Life Sciences, 324, 121722, 10.1016/j.lfs.2023.121722 37100379

[jev270020-bib-0135] Savitski, M M. , Mathieson, T. , Zinn, N. , Sweetman, G. , Doce, C. , Becher, I. , Pachl, F. , Kuster, B. , & Bantscheff, M. (2013). Measuring and managing ratio compression for accurate iTRAQ/TMT quantification. Journal of Proteome Research, 12, 3586–3598. 10.1021/pr400098r 23768245

[jev270020-bib-0136] Scheerlinck, E. , Dhaenens, M. , Van Soom, A. , Peelman, L. , De Sutter, P. , Van Steendam, K. , & Deforce, D. (2015). Minimizing technical variation during sample preparation prior to label‐free quantitative mass spectrometry. Analytical Biochemistry, 490, 14–19. 10.1016/j.ab.2015.08.018 26302362

[jev270020-bib-0137] Schmudlach, A. , Felton, J. , Cipolla, C. , Sun, L. , Kennedy, R T. , & Dovichi, N J. (2016). Sample preparation protocol for bottom‐up proteomic analysis of the secretome of the islets of Langerhans. The Analyst, 141, 1700–1706. 10.1039/C5AN02265G 26863548 PMC4764456

[jev270020-bib-0138] Searle, B C. , Pino, L K. , Egertson, J D. , Ting, Y S. , Lawrence, R T. , Maclean, B X. , Villén, J. , & Maccoss, M J. (2018). Chromatogram libraries improve peptide detection and quantification by data independent acquisition mass spectrometry. Nature Communications, 9, 10.1038/s41467-018-07454-w PMC6277451 30510204

[jev270020-bib-0139] Shen, S. , Tu, C. , Shen, H. , Li, J. , Frangou, C. , Zhang, J. , & Qu, J. (2023). Comparative proteomics analysis of exosomes identifies key pathways and protein markers related to breast cancer metastasis. International Journal of Molecular Sciences, 24(4), 4033. 10.3390/ijms24044033 36835443 PMC9967130

[jev270020-bib-0140] Shevchenko, A. , Tomas, H. , Havli, J. , Olsen, J. V. , & Mann, M. (2006). In‐gel digestion for mass spectrometric characterization of proteins and proteomes. Nature Protocols, 1, 2856–2860. 10.1038/nprot.2006.468 17406544

[jev270020-bib-0141] Sielaff, M. , Kuharev, J. , Bohn, T. , Hahlbrock, J. , Bopp, T. , Tenzer, S. , & Distler, U. (2017). Evaluation of FASP, SP3, and iST protocols for proteomic sample preparation in the low microgram range. Journal of Proteome Research, 16, 4060–4072. 10.1021/acs.jproteome.7b00433 28948796

[jev270020-bib-0142] Skalnikova, H. K. , Bohuslavova, B. , Turnovcova, K. , Juhasova, J. , Juhas, S. , Rodinova, M. , & Vodicka, P. (2019). Isolation and characterization of small extracellular vesicles from porcine blood plasma, cerebrospinal fluid, and seminal plasma. Proteomes, 7(2), 17. 10.3390/proteomes7020017 31027284 PMC6630935

[jev270020-bib-0143] Skoczylas, Ł. , Gawin, M. , Fochtman, D. , Widłak, P. , Whiteside, T. L. , & Pietrowska, M. (2024). Immune capture and protein profiling of small extracellular vesicles from human plasma. Proteomics, 24(11), e2300180. 10.1002/pmic.202300180 37713108 PMC11046486

[jev270020-bib-0144] Smith, I R. , Eng, J K. , Barente, A S. , Hogrebe, A. , Llovet, A. , Rodriguez‐Mias, R A. , & Villén, J. (2022). Coisolation of peptide pairs for peptide identification and MS/MS‐based quantification. Analytical Chemistry, 94, 15198–15206. 10.1021/acs.analchem.2c01711 36306373 PMC9851627

[jev270020-bib-0145] Smolarz, M. , Skoczylas, Ł. , Gawin, M. , Krzyżowska, M. , Pietrowska, M. , & Widłak, P. (2022). Radiation‐induced bystander effect mediated by exosomes involves the replication stress in recipient cells. International Journal of Molecular Sciences, 23, 4169, 10.3390/ijms23084169 35456987 PMC9029583

[jev270020-bib-0146] Soares Martins, T. , Catita, J. , Martins Rosa, I. , A B Da Cruz E Silva, O. , & Henriques, A. G. (2018). Exosome isolation from distinct biofluids using precipitation and column‐based approaches. PLOS ONE, 13, e0198820. 10.1371/journal.pone.0198820 29889903 PMC5995457

[jev270020-bib-0147] Sódar, B. W. , Kittel, Á. , Pálóczi, K. , Vukman, K. V. , Osteikoetxea, X. , Szabó‐Taylor, K. , Németh, A. , Sperlágh, B. , Baranyai, T. , Giricz, Z. , Wiener, Z. , Turiák, L. , Drahos, L. , Pállinger, É. , Vékey, K. , Ferdinandy, P. , Falus, A. , & Buzás, E. I. (2016). Low‐density lipoprotein mimics blood plasma‐derived exosomes and microvesicles during isolation and detection. Scientific Reports, 6, 24316. 10.1038/srep24316 27087061 PMC4834552

[jev270020-bib-0148] Soloveva, N. , Novikova, S. , Farafonova, T. , Tikhonova, O. , & Zgoda, V. (2023). Proteomic signature of extracellular vesicles associated with colorectal cancer. Molecules, 28, 4227. 10.3390/molecules28104227 37241967 PMC10222552

[jev270020-bib-0149] Stepath, M. , Zülch, B. , Maghnouj, A. , Schork, K. , Turewicz, M. , Eisenacher, M. , Hahn, S. , Sitek, B. , & Bracht, T. (2020). Systematic comparison of label‐free, SILAC, and TMT techniques to study early adaption toward inhibition of EGFR signaling in the colorectal cancer cell line DiFi. Journal of Proteome Research, 19, 926–937. 10.1021/acs.jproteome.9b00701 31814417

[jev270020-bib-0150] Subedi, P. , Schneider, M. , Philipp, J. , Azimzadeh, O. , Metzger, F. , Moertl, S. , Atkinson, M J. , & Tapio, S. (2019). Comparison of methods to isolate proteins from extracellular vesicles for mass spectrometry‐based proteomic analyses. Analytical Biochemistry, 584, 113390. 10.1016/j.ab.2019.113390 31401005

[jev270020-bib-0151] Stamatia Rontogianni . Synadaki, E. , Li, B. , Liefaard, M C. , Lips, E H. , Wesseling, J. , Wu, W. , & Altelaar, M. . (2019). Proteomic profiling of extracellular vesicles allows for human breast cancer subtyping. Communications Biology, 2, 325. 10.1038/s42003-019-0570-8 31508500 PMC6722120

[jev270020-bib-0152] Thermo Scientific . https://assets.thermofisher.com/TFS‐Assets/LSG/Application‐Notes/TR0068‐Protein‐assay‐compatibility.pdf

[jev270020-bib-0153] Thermo Fisher Scientific Inc . Xcalibur. Proteome Discoverer. User Guide.; XCALI‐97232 Revision A; 2008.

[jev270020-bib-0154] Théry, C. , Amigorena, S. , Raposo, G. , & Clayton, A. (2006). Isolation and characterization of exosomes from cell culture supernatants and biological fluids. *Current Protocols in Cell Biology*. 10.1002/0471143030.cb0322s30 18228490

[jev270020-bib-0155] Thompson, A. , Schäfer, J. , Kuhn, K. , Kienle, S. , Schwarz, J. , Schmidt, G. , Neumann, T. , & Hamon, C. (2003). Tandem mass tags: A novel quantification strategy for comparative analysis of complex protein mixtures by MS/MS. Analytical Chemistry, 75, 1895–1904. 10.1021/ac0262560 12713048

[jev270020-bib-0156] Tian, W. , Shi, D. , Zhang, Y. , Wang, H. , Tang, H. , Han, Z. , Wong, C C. L. , Cui, L. , Zheng, J. , & Chen, Y. (2024). Deep proteomic analysis of obstetric antiphospholipid syndrome by DIA‐MS of extracellular vesicle enriched fractions. Communications Biology, 7, 10.1038/s42003-024-05789-3 PMC10789860 38225453

[jev270020-bib-0157] Torres, A. , Bernardo, L. , Sánchez, C. , Morato, E. , Solana, J. C. , & Carrillo, E. (2024). Comparing the proteomic profiles of extracellular vesicles isolated using different methods from long‐term stored plasma samples. Biological Procedures Online, 26(1), 18. 10.1186/s12575-024-00243-4 38898416 PMC11188224

[jev270020-bib-0158] Tsuno, H. , Arito, M. , Suematsu, N. , Sato, T. , Hashimoto, A. , Matsui, T. , Omoteyama, K. , Sato, M. , Okamoto, K. , Tohma, S. , Kurokawa, M S. , & Kato, T. (2018). A proteomic analysis of serum‐derived exosomes in rheumatoid arthritis. BMC Rheumatology, 2, 35. 10.1186/s41927-018-0041-8 30886985 PMC6390805

[jev270020-bib-0159] Urinary Exosome Protein Database . https://esbl.nhlbi.nih.gov/UrinaryExosomes/

[jev270020-bib-0160] Urzì, O. , Olofsson Bagge, R. , & Crescitelli, R. (2022). The dark side of foetal bovine serum in extracellular vesicle studies. *Journal of Extracellular Vesicles*, *11*. 10.1002/jev2.12271 PMC9549727 36214482

[jev270020-bib-0161] Uszkoreit, J. , Barkovits, K. , Pacharra, S. , Pfeiffer, K. , Steinbach, S. , Marcus, K. , & Eisenacher, M. (2022). Dataset containing physiological amounts of spike‐in proteins into murine C2C12 background as a ground truth quantitative LC‐MS/MS reference. Data in Brief, 43, 108435. 10.1016/j.dib.2022.108435 35845101 PMC9283871

[jev270020-bib-0162] Välikangas, T. , Suomi, T. , & Elo, L. L. (2018). A systematic evaluation of normalization methods in quantitative label‐free proteomics. Briefings in Bioinformatics, 19(1), 1–11. 10.1093/bib/bbw095 27694351 PMC5862339

[jev270020-bib-0163] Vallejos, P A. , Fuller, R N. , Kabagwira, J. , Kwong, M. L. , Gonda, A. , Mcmullen, J R. W. , Le, N. , Selleck, M J. , Miller, L D. , Perry, C C. , Senthil, M. , & Wall, N R. (2023). Exosomal proteins as a source of biomarkers in colon cancer‐derived peritoneal carcinomatosis—A pilot study. PROTEOMICS—Clinical Applications, 17(2), e2100085. 10.1002/prca.202100085 36217952

[jev270020-bib-0164] Varnavides, G. , Madern, M. , Anrather, D. , Hartl, N. , Reiter, W. , & Hartl, M. (2022). In search of a universal method: A comparative survey of bottom‐up proteomics sample preparation methods. Journal of Proteome Research, 21, 2397–2411. 10.1021/acs.jproteome.2c00265 36006919 PMC9552232

[jev270020-bib-0165] Wang, J.‐H. , Choong, W.‐K. , Chen, C.‐T. , & Sung, T.‐Y. (2022). Calibr improves spectral library search for spectrum‐centric analysis of data independent acquisition proteomics. Scientific Reports, 12, 2045. 10.1038/s41598-022-06026-9 35132134 PMC8821666

[jev270020-bib-0166] Wang, Z. G. , He, Z. Y. , Liang, S. , Yang, Q. , Cheng, P. , & Chen, A. M. (2020). Comprehensive proteomic analysis of exosomes derived from human bone marrow, adipose tissue, and umbilical cord mesenchymal stem cells. Stem Cell Research & Therapy, 11(1), 511. 10.1186/s13287-020-02032-8 33246507 PMC7694919

[jev270020-bib-0167] Wang, Z. , Hill, S. , Luther, J M. , Hachey, D L. , & Schey, K L. (2012). Proteomic analysis of urine exosomes by multidimensional protein identification technology (MudPIT). Proteomics, 12, 329–338. 10.1002/pmic.201100477 22106071 PMC3517144

[jev270020-bib-0168] Wang, Z. , Mülleder, M. , Batruch, I. , Chelur, A. , Textoris‐Taube, K. , Schwecke, T. , Hartl, J. , Causon, J. , Castro‐Perez, J. , Demichev, V. , Tate, S. , & Ralser, M. (2022). High‐throughput proteomics of nanogram‐scale samples with Zeno SWATH MS. eLife, 11, e83947. 10.7554/eLife.83947 36449390 PMC9711518

[jev270020-bib-0169] Welsh, J. A. , Goberdhan, D. C. I. , O'driscoll, L. , Buzas, E. I. , Blenkiron, C. , Bussolati, B. , Cai, H. , Di Vizio, D. , Driedonks, T. A. P. , Erdbrügger, U. , Falcon‐Perez, J. M. , Fu, Q.‐L. , Hill, A. F. , Lenassi, M. , Lim, S. K. , Mahoney, M. G. , Mohanty, S. , Möller, A. , Nieuwland, R. , … Witwer, K. W. (2024). Minimal information for studies of extracellular vesicles (MISEV2023): From basic to advanced approaches. Journal of Extracellular Vesicles, 13, 1–84. 10.1002/jev2.12404 PMC10850029 38326288

[jev270020-bib-0170] Wierenga, S K. , Zocher, M J. , Mirus, M M. , Conrads, T P. , Goshe, M B. , & Veenstra, T D. (2002). A method to evaluate tryptic digestion efficiency for high‐throughput proteome analyses. Rapid Communications in Mass Spectrometry, 16(14), 1404–1408. 10.1002/rcm.729 12112621

[jev270020-bib-0171] Winter, D. , & Steen, H. (2011). Optimization of cell lysis and protein digestion protocols for the analysis of HeLa S3 cells by LC‐MS/MS. Proteomics, 11, 4726–4730. 10.1002/pmic.201100162 22002805

[jev270020-bib-0172] Wiśniewski, J R. , Zougman, A. , & Mann, M. (2009). Combination of FASP and StageTip‐based fractionation allows in‐depth analysis of the hippocampal membrane proteome. Journal of Proteome Research, 8, 5674–5678. 10.1021/PR900748N/SUPPL_FILE/PR900748N_SI_004.XLS 19848406

[jev270020-bib-0173] Wiśniewski, J. R. (2018). Filter‐Aided Sample Preparation for Proteome Analysis, in Microbial proteomics (1841), Springer, pp. 3–10. 10.1007/978-1-4939-8695-8_1 30259475

[jev270020-bib-0174] Wiśniewski, J. R. (2019). Filter aided sample preparation—A tutorial. Analytica Chimica Acta, 1090, 23–30. 10.1016/j.aca.2019.08.032 31655642

[jev270020-bib-0175] Wiśniewski, J. R. , & Mann, M. (2016). A proteomics approach to the protein normalization problem: Selection of unvarying proteins for MS‐based proteomics and western blotting. Journal of Proteome Research, 15, 2321–2326. 10.1021/acs.jproteome.6b00403 27297043

[jev270020-bib-0176] Wolf, M. , Poupardin, R. W. , Ebner‐Peking, P. , Andrade, A. C. , Blöchl, C. , Obermayer, A. , Gomes, F. G. , Vari, B. , Maeding, N. , Eminger, E. , Binder, H.‐M. , Raninger, A. M. , Hochmann, S. , Brachtl, G. , Spittler, A. , Heuser, T. , Ofir, R. , Huber, C. G. , Aberman, Z. , & Strunk, D. (2022). A functional corona around extracellular vesicles enhances angiogenesis, skin regeneration and immunomodulation. Journal of Extracellular Vesicles, 11, 10.1002/jev2.12207 PMC8994701 35398993

[jev270020-bib-0177] Wong, C.‐H. , & Chen, Y.‐C. (2019). Clinical significance of exosomes as potential biomarkers in cancer. World Journal of Clinical Cases, 7(2), 171–190. 10.12998/wjcc.v7.i2.171 30705894 PMC6354096

[jev270020-bib-0178] Wu, C. C. , Tsantilas, K. A. , Park, J. , Plubell, D. , Sanders, J. A. , Naicker, P. , Govender, I. , Buthelezi, S. , Stoychev, S. , Jordaan, J. , Merrihew, G. , Huang, E. , Parker, E. D. , Riffle, M. , Hoofnagle, A. N. , Noble, W. S. , Poston, K. L. , Montine, T. J. , & MacCoss, M. J. (2024). Mag‐Net: Rapid enrichment of membrane‐bound particles enables high coverage quantitative analysis of the plasma proteome. 10.1101/2023.06.10.544439

[jev270020-bib-0179] Wu, Y. , Wu, M. , He, G. , Zhang, X. , Li, W. , Gao, Y. , Li, Z. , Wang, Z. , & Zhang, C. (2012). Glyceraldehyde‐3‐phosphate dehydrogenase: A universal internal control for Western blots in prokaryotic and eukaryotic cells. Analytical Biochemistry, 423, 15–22. 10.1016/j.ab.2012.01.012 22326796

[jev270020-bib-0180] Yang, C. , Zhang, M. , Sung, J. , Wang, L. , Jung, Y. , & Merlin, D. (2020). Isolation and characterization of exosomes from mouse feces. Bio‐protocol, 10(8), e3584. 10.21769/BioProtoc.3584 32440530 PMC7241525

[jev270020-bib-0181] Yang, D. , Zhang, W. , Zhang, H. , Zhang, F. , Chen, L. , Ma, L. , Larcher, L. M. , Chen, S. , Liu, N. , Zhao, Q. , Tran, P. H. L. , Chen, C. , Veedu, R. N. , & Wang, T. (2020). Progress, opportunity, and perspective on exosome isolation—Efforts for efficient exosome‐based theranostics. Theranostics, 10(8), 3684–3707. 10.7150/thno.41580 32206116 PMC7069071

[jev270020-bib-0182] Yang, F. , Shen, Y. , Camp, D. G. , & Smith, R. D. (2012). High pH reversed‐phase chromatography with fraction concatenation as an alternative to strong‐cation exchange chromatography for two‐dimensional proteomic analysis. Expert Review of Proteomics, 9, 129–134. 10.1586/epr.12.15 22462785 PMC3337716

[jev270020-bib-0183] Yu, D. , Li, Y. , Wang, M. , Gu, J. , Xu, W. , Cai, H. , Fang, X. , & Zhang, X. (2022). Exosomes as a new frontier of cancer liquid biopsy. Molecular Cancer, 21(1), 56. 10.1186/s12943-022-01509-9 35180868 PMC8855550

[jev270020-bib-0184] Yu, K. , Xiao, K. , Sun, Q. Q. , Liu, R. F. , Huang, L. F. , Zhang, P. F. , Xu, H. Y. , Lu, Y. Q. , & Fu, Q. (2023). Comparative proteomic analysis of seminal plasma exosomes in buffalo with high and low sperm motility. BMC Genomics, 24(1), 8. 10.1186/s12864-022-09106-2 36624393 PMC9830767

[jev270020-bib-0185] Yu, Z. , Zhao, C. , Hu, S. , Zhang, H. , Li, W. , Zhang, R. , Luo, Q. , & Yang, H. (2021). MALDI‐MS‐based biomarker analysis of extracellular vesicles from human lung carcinoma cells. RSC Advances, 11(41), 25375–25380. 10.1039/d1ra04305f 35478925 PMC9037017

[jev270020-bib-0186] Zarovni, N. , Corrado, A. , Guazzi, P. , Zocco, D. , Lari, E. , Radano, G. , Muhhina, J. , Fondelli, C. , Gavrilova, J. , & Chiesi, A. (2015). Integrated isolation and quantitative analysis of exosome shuttled proteins and nucleic acids using immunocapture approaches. Methods, 10.1016/j.ymeth.2015.05.028 26044649

[jev270020-bib-0187] Zebrowska, A. , Jelonek, K. , Mondal, S. , Gawin, M. , Mrowiec, K. , Widłak, P. , Whiteside, T. , & Pietrowska, M. (2022). Proteomic and metabolomic profiles of T cell‐derived exosomes isolated from human plasma. Cells, 11(12), 1965. 10.3390/cells11121965 35741093 PMC9222142

[jev270020-bib-0188] Zhang, F. , Ge, W. , Huang, L. , Li, D. , Liu, L. , Dong, Z. , Xu, L. , Ding, X. , Zhang, C. , Sun, Y. , A, J. , Gao, J. , & Guo, T. (2023). A comparative analysis of data analysis tools for data‐independent acquisition mass spectrometry. Molecular & Cellular Proteomics, 22(9), 100623. 10.1016/j.mcpro.2023.100623 37481071 PMC10458344

[jev270020-bib-0189] Zhang, J. , Xin, L. , Shan, B. , Chen, W. , Xie, M. , Yuen, D. , Zhang, W. , Zhang, Z. , Lajoie, G. A. , & Ma, B. (2012). PEAKS DB: De novo sequencing assisted database search for sensitive and accurate peptide identification. Molecular & Cellular Proteomics, 11(4), M111.010587. 10.1074/mcp.M111.010587 PMC3322562 22186715

[jev270020-bib-0190] Zhang, W. , Ou, X. , & Wu, X. (2019). Proteomics profiling of plasma exosomes in epithelial ovarian cancer: A potential role in the coagulation cascade, diagnosis and prognosis. International Journal of Oncology, 54(5), 1719–1733. 10.3892/ijo.2019.4742 30864689 PMC6438431

[jev270020-bib-0191] Zhang, Y. , Wu, J. L. Y. , Lazarovits, J. , & Chan, W. C. W. (2020). An analysis of the binding function and structural organization of the protein corona. Journal of the American Chemical Society, 142(19), 8827–8836. 10.1021/jacs.0c01853 32293877

[jev270020-bib-0192] Zhao, L. , Cong, X. , Zhai, L. , Hu, H. , Xu, J.‐Y. , Zhao, W. , Zhu, M. , Tan, M. , & Ye, B.‐C. (2020). Comparative evaluation of label‐free quantification strategies. Journal of Proteomics, 215, 103669. 10.1016/j.jprot.2020.103669 31987925

[jev270020-bib-0193] Zheng, X. , Xu, K. , Zhou, B. , Chen, T. , Huang, Y. , Li, Q. , Wen, F. , Ge, W. , Wang, J. , Yu, S. , Sun, L. , Zhu, L. , Liu, W. , Gao, H. , Yue, L. , Cai, X. , Zhang, Q. , Ruan, G. , Zhu, T. , … Zheng, S. (2020). A circulating extracellular vesicles‐based novel screening tool for colorectal cancer revealed by shotgun and data‐independent acquisition mass spectrometry. Journal of Extracellular Vesicles, 9(1), 1750202. 10.1080/20013078.2020.1750202 32363013 PMC7178829

[jev270020-bib-0194] Zhou, B. , Xu, K. , Zheng, X. , Chen, T. , Wang, J. , Song, Y. , Shao, Y. , & Zheng, S. (2020). Application of exosomes as liquid biopsy in clinical diagnosis. Signal Transduction and Targeted Therapy, 5(1), 144. 10.1038/s41392-020-00258-9 32747657 PMC7400738

[jev270020-bib-0195] Zhu, S. , Xing, C. , Li, R. , Cheng, Z. , Deng, M. , Luo, Y. , Li, H. , Zhang, G. , Sheng, Y. , Peng, H. , & Wang, Z. (2022). Proteomic profiling of plasma exosomes from patients with B‐cell acute lymphoblastic leukemia. Scientific Reports, 12, 11975. 10.1038/s41598-022-16282-4 35831551 PMC9279438

[jev270020-bib-0196] Zhu, Y. , Pick, H. , Gasilova, N. , Li, X. , Lin, T.‐E. , Laeubli, H. P. , Zippelius, A. , Ho, P.‐C. , & Girault, H. H. (2019). MALDI detection of exosomes: A potential tool for cancer studies. Chem, 5(5), 1318–1336. 10.1016/j.chempr.2019.04.007

[jev270020-bib-0197] Zougman, A. , Wilson, J. P. , & Banks, R. E. (2020). A simple serum depletion method for proteomics analysis. BioTechniques, 69, 148–151. 10.2144/btn-2020-0017 32372655

